# Macroevolutionary Patterns in the Aphidini Aphids (Hemiptera: Aphididae): Diversification, Host Association, and Biogeographic Origins

**DOI:** 10.1371/journal.pone.0024749

**Published:** 2011-09-15

**Authors:** Hyojoong Kim, Seunghwan Lee, Yikweon Jang

**Affiliations:** 1 Division of EcoScience and Research Institute of EcoScience, Ewha Womans University, Seoul, Republic of Korea; 2 Department of Agricultural Biotechnology, Research Institute for Agriculture and Life Science, Seoul National University, Seoul, Republic of Korea; Field Museum of Natural History, United States of America

## Abstract

**Background:**

Due to its biogeographic origins and rapid diversification, understanding the tribe Aphidini is key to understanding aphid evolution. Major questions about aphid evolution include origins of host alternation as well as age and patterns of diversification in relation to host plants. To address these questions, we reconstructed the phylogeny of the Aphidini which contains *Aphis*, the most diverse genus in the family. We used a combined dataset of one nuclear and four mitochondrial DNA regions. A molecular dating approach, calibrated with fossil records, was used to estimate divergence times of these taxa.

**Principal Findings:**

Most generic divergences in Aphidini occurred in the Middle Tertiary, and species-level divergences occurred between the Middle and Late Tertiary. The ancestral state of host use for Aphidini was equivocal with respect to three states: monoecy on trees, heteroecy, and monoecy on grasses. The ancestral state of Rhopalosiphina likely included both heteroecy and monoecy, whereas that of Aphidina was most likely monoecy. The divergence times of aphid lineages at the generic or subgeneric levels are close to those of their primary hosts. The species-level divergences in aphids are consistent with the diversification of the secondary hosts, as a few examples suggest. The biogeographic origin of Aphidini as a whole was equivocal, but the major lineages within Aphidina likely separated into Nearctic, Western Palearctic, and Eastern Palearctic regions.

**Conclusions:**

Most generic divergences in Aphidini occurred in the Middle Tertiary when primary hosts, mainly in the Rosaceae, were diverging, whereas species-level divergences were contemporaneous with diversification of the secondary hosts such as Poaceae in the Middle to Late Tertiary. Our results suggest that evolution of host alternation within Aphidini may have occurred during the Middle Tertiary (Oligocene) when the secondary hosts emerged.

## Introduction

The biology of aphids features some characteristics unusual in the animal kingdom, namely: polyphenism, alternation of sexual and asexual reproduction, and host alternation [1], [2], [3]. Evolution of these unusual characteristics is thought to be related to aphids' intricate ecological associations and evolutionary co-diversification with their their host plants [4], [5]. Although there is ample evidence of co-diversification of insects and their host plants across various taxa [6], [7], [8], [9], [10], major macroevolutionary patterns of co-diversification between them including age, patterns of diversification, and biogegraphic origins often remain unclear [10]. For example, Lopez-Vaamonde et al. [11] proposed three hypotheses of temporal relationship between plant and insect diversifications: cospeciation, fast colonization, and delayed colonization. The cospeciation hypothesis is basically synchronized coevolution between phytophagous insects and their host plants, leading to congruent phylogenies and no time lag in diversifications between them [11], [12]. In both of the delayed colonization scenarios, phytophagous insects do not coevolve but instead colonize host plants that have already diversified in both fast and delayed colonization hypotheses [7], [13]. Depending on the magnitude of evolutionary innovations required for using newly-diversified plants as resources, colonization may be fast or delayed [7], [11], [13].

Aphids are phloem-feeding insects, capable of infesting more than 40 plant families worldwide [2], [3], [14]. Based on fossil evidence and phylogenies, the ancestral aphids are hypothesized to have lived on woody host plants and reproduced sexually throughout the season [4], [15], [16]. Early in their evolution, aphids established parthenogenesis for their reproduction, as is found in all extant aphid taxa [4], [5]. Typically, aphids undergo a series of all-female parthenogenetic generations, followed by a single generation of sexual reproduction [5]. This is called cyclical parthenogenesis, or holocycly [5]. Some aphids exhibit anholocycly in which the sexual generation is eliminated entirely; it is hypothesized that anholocycly originated from holocycly based on loss of the sexual phase [5].

Another unusual feature of aphid evolution is the life cycle in relation to host plant use [1], [5]. Monoecious aphids use the same type of host plants throughout their entire life cycles, whereas heteroecious aphids display host alternation between two distantly-related host plants, typically with the primary woody plants for sexual reproduction and the secondary herbaceous hosts for the parthenogenetic segment of a life cycle [4], [5]. Therefore, all heteroecious aphids are holocyclic. There are in general three types of life cycle in extant aphids: (1) monoecy on trees, (2) heteroecy, and (3) monoecy on grasses [4], [5]. Monoecy on trees is assumed to be the ancestral state for the family. Heteroecy is a more recently evolved state, in which a secondary host is acquired and the generations alternate host plants. Monoecy on grasses is then thought to have been derived through loss of the primary host tree species [4], [5]. Less than 15% of aphids in the family Aphididae exhibit host alternation [4], [5], [17]. Heteroecy is most likely to have evolved in the Tertiary [4], [5], [16]. Contrary to the classical view of host alternation as a plesiomorphic trait inherited from a common Aphididae ancestor [18], [19], Moran [4], [20] suggested multiple gains within the subfamily Aphidinae. Later, based on a molecular phylogeny of Aphidinae, von Dohlen et al. [21] suggested that host alternation evolved twice: arising independently in both the tribes Aphidini and Macrosiphini. However, it still remains unclear when and how the different origins of host alternation arose for these groups, as their divergence times have never been estimated by a firm phylogenetic framework or compared with those of their host plants.

Approximately 5,000 described species of aphids belong to the family Aphididae (Hemiptera) [17], which may have diverged from the common ancestor of Adelgidae and Phylloxeridae in the Cretaceous [16], [22]. Aphididae is divided into 27 subfamilies based on phenotypic, life cycle-specific, and host-specific variations [3], [17]. Of the subfamilies, Aphidinae, which includes numerous agricultural pests, is the most diverse in the temperate regions of the Northern Hemisphere and subtropical regions [17], [23]. Most modern taxa of Aphidinae likely diversified during the Tertiary [15], [16]. Based on fossil records, at least 50% of the extant species of Aphidinae may have originated in the Middle to Late Tertiary [15], [19]. The tribes Aphidini and Macrosiphini constitute Aphidinae, which has a sister relationship with the relatively small subfamily, Pterocommatinae. The tribe Aphidini contains more than 800 valid species, these aphids are relatively small and morphologically simple [14], [17], [24]. In a proposed alternative classification, Aphidini has been suggested as primitive to Macrosiphini, if Pterocommaninae and Macrosiphini form a clade [21]. In addition, Aphidini is considered to be a possible origin of Aphidinae, because this tribe is the only group that contains species indigenous to the Southern Hemisphere [21], [25]. Aphidini is subdivided into two monophyletic subtribes, Aphidina and Rhopalosiphina [26]. The subtribe Aphidina contains the most species-rich genus, *Aphis*, whose rapid diversification may exemplify the evolutionary patterns of extant aphids [27]. Therefore, knowledge of taxon ages and patterns of diversification in Aphidini are critical to our understanding of aphid evolution [21].

We reconstructed the phylogeny of the tribe Aphidini and close relatives using DNA sequence data from one nuclear and four mitochondrial genes. Furthermore, we estimated divergence times using a molecular dating approach. Information generated in this study will be critical for understanding ages and patterns of diversification, origins of host alternation [16], [21], and biogeographic origins in the aphids [14].

## Methods

### Ethical treatment of animals

Ethical approval was not required for work with the aphids, the subjects in this study, because aphids are invertebrates, and they are not listed as endangered species. Aphids are abundant almost everywhere in their natural ranges.

### Taxon sampling and outgroup selection

A total of 80 ingroup species (59 Aphidina, 12 Rhopalosiphina, seven Macrosiphini, and two Pterocommatinae spp.) and seven outgroup species (two Hormaphidinae, one Lachninae, two Eriosomatinae, one Adelgidae, and one Phylloxeridae spp.) were used in this study (Table S1). We collected 46 species samples in the central and southern regions of the Korean Peninsula between 2003 and 2007, and, when available, used some sequences from previous studies [26], [27], [28]. DNA sequences of the ingroup species in Nearctic, European, and Australasian regions were obtained from GenBank (Table S1). The rest of the Aphidini sequences used in this study were derived from von Dohlen & Teulon [25], Turcinaviciene et al. [29], and Coeur d'acier et al. [30], to ensure representation of phylogenetically important taxa in each region. The sequences of the outgroup species, *Adelges cooleyi*, *Phylloxera* sp., *Hamamelistes spinosus*, *Melaphis rhois*, and *Schlechtendalia chinensis*, were also obtained from GenBank to get calibration points for dating analysis (Table S1, Figure S1) [16], [21], [22], [23], [29], [31], [32], [33].

Within Aphidina, most species were sampled from the genus *Aphis*, which consists of four main species-groups (*craccivora*, *fabae*, *gossypii*, and *spiraecola*), as well as from three other major subgenera (*Bursaphis*, *Protaphis*, *Toxopterina*). Two undescribed heteroecious species, *Aphis* sp.1 and sp.2 ex *Rhamnus* were included [28]. Two different types (type 1 and 2) of *A. gossypii* were collected from *Rhamnus*, its primary host; these were genetically different from other secondary host associated types, which were also included [28]. *Toxoptera aurantii* was included as a representative taxon characterized by a complete anholocyclic life [34]. Four major genera, *Hyalopterus*, *Melanaphis*, *Rhopalosiphum*, and *Schizaphis*, were included within Rhopalosiphina. *Aphis cottieri* Carver, *A. healyi* Cottier, *Casimira* sp., *Euschizaphis* sp.1, *Euschizaphis* sp.2, *Paradoxaphis aristoteliae* Sunde, and *P. plagianthi* Eastop, indigenous to the Southern Hemisphere, were included in order to determine whether Aphidinae or Aphidini originated there [25]. One sister clade of Aphidini, Macrosiphini, was represented by six genera (*Acyrthosiphon*, *Brevicoryne*, *Cryptosiphum*, *Lipaphis*, *Megoura*, and *Myzus*), which acted as representatives of two monophyletic lineages, Dactynotines and Myzines. For the other sister clade of Aphidini,*Pterocomma*+*Cavariella* were selected for construction of the expected clade of Pterocommatinae+*Cavariella* (P-C group), which had emerged as a monophyletic group in a previous phylogeny [21]. Two outgroups were selected at different taxonomic levels in order to set the calibration points precisely as well as to obtain reliable diversification times of Aphidinae corresponding to previous phylogenetic studies [16], [22], [35]. The first outgroup for fixing the calibration point diverging from the family Aphididae was the clade of Adelgidae+Phylloxeridae (*Adelges cooleyi* (Gillette) and *Phylloxera* sp.). The second outgroup for constraining the divergence point of the Aphididae crown clade consisted of three relative or distant subfamilies, Lachninae (*Cinara longipennis* (Matsumura)), Hormaphidinae (*Hamamelistes spinosus* Shimer and *Nipponaphis coreanus* (Paik)), Eriosomatinae (*Melaphis rhois* (Fitch), and *Schlechtendalia chinensis* (Bell)). Due to the rapid diversification of Aphididae subfamilies during the Cretaceous [16], it is still uncertain which group within Aphididae is the most basal lineage. Ortiz-Rivas and Martinez-Torres [36] recently reported that Lachninae is the most basal lineage within Aphididae, but uncertainty remains due to sampling bias and constrained nodes. In contrast, Heie [15], [19] suggested that Hormaphidinae and Eriosomatinae have more plesiomorphic morphological characters (e.g., shapes of antenna, secondary rhinaria, abdomen, and wing venation) than Lachninae. Therefore, three different subfamilies (Hormaphidinae, Lachninae, and Eriosomatinae) were used for calibrating the age of the Aphididae, in order to avoid uncertainties in the current phylogeny (von Dohlen and Moran, 2000; Ortiz-Rivas and Martinez-Torres, 2009). Two eriosomatids whose fossil and host plant data are available for divergence time calculation [37], [38] were also used as a calibration point for the molecular dating analysis.

### DNA sequencing and alignment

Total genomic DNA was extracted from single individuals using a DNeasy® Blood & Tissue Kit (QIAGEN, Inc., Düsseldorf) following the manufacturer's protocol. The primers for PCR amplification are listed in Table S2. LCO1490f and HCO2198 [39] were used to amplify partial cytochrome *c* oxidase I (COI). Primers 2993+ [40] and A3772 [41] were used to amplify partial tRNA-leucine+cytochrome c oxidase II (tRNA/COII). Primer F18 coupled with R18 [42] or CB2 [43] were used to amplify cytochrome *b* (CytB). Primers 12Sai [44] and 1473 [16] were used to amplify partial 12S rRNA+tRNA-valine+16S rRNA (12S/16S). Three primers, 12Sfr (a reverse of 12Sfi [45]), 1470a, and 1472 [16], were used as internal primers for sequencing. Primer EF3 coupled with EF2 [46] or EF6 [47] was used to amplify elongation factor 1 alpha EF1α.

DNA fragments were amplified using AccuPower® PCR PreMix (BIONEER, Corp., Daejeon) in 20 µl reaction mixtures containing 0.4 µM of each primer, 20 µM of dNTPs, 20 µM of MgCl_2_, and 0.05 µg of genomic DNA template. PCR was performed using a GS482 thermo-cycler (Gene Technologies, Ltd., Essex) according to the following procedure: initial denaturation at 95°C for 5 min, followed by 34 cycles at 95°C for 30 sec; annealing temperature (43–45°C depending on the primer sets) for 30–50 sec; extension at 72°C for 30–60 sec, and final extension at 72°C for 5 min. The primer-specific annealing temperatures of each primer set were 43°C for COI, 42–45°C for tRNA/COII, 43–47°C for CytB, 48.5°C for 12S/16S, and 53–58°C for EF1α. PCR products were visualized by electrophoresis on a 1.5% agarose gel. A single band was observed, purified using a QIAquick® PCR purification kit (QIAGEN, Inc.), and then sequenced directly using an automated sequencer (ABI Prism® 3730 XL DNA Analyzer). The sequences generated in this study were all deposited in GenBank (Table S1).

Raw sequences were examined and corrected using SeqMan™II (version 7.1.0, 2006; DNAstar™). All DNA sequences for each fragment were aligned using Clustal X version 2.0.11 ([48]; with default settings). The intron splicing junctions of nuclear EF1α sequences were identified and removed using MEGA 4.0 [49]. Ambiguous sites in 12S/16S containing the most gaps were removed using GBLOCKS 0.91b ([50]; default settings except for the allowed gap option where ‘with half’ was used). Uncorrected *P*-distances, number of substitutions, Transition (Ti)/Transversion (Tv) ratio, and nucleotide compositions for COI, tRNA/COII, CytB, and EF1α were also obtained using MEGA.

### Phylogenetic analysis

Maximum parsimony (MP) analyses were performed with PAUP*4.0b10 [51] using a heuristic search procedure with 1000 random additions of sequences and 10 trees held at each pseudoreplicate by following the TBR branch swapping method. All characters were treated as unordered and equally weighted for MP analysis. Bootstrapping was conducted using 1000 replicates under the heuristic search procedure with 10 random-addition sequences. A partition-homogeneity test [52], as implemented in PAUP*, was performed using a heuristic search with 1000 replicates for significant phylogenetic analysis of the four mtDNA regions and EF1α in two ways: i) individual mtDNA region vs EF1α, ii) combined mtDNA dataset vs EF1α. Taxa missing data for any dataset were automatically removed from the test.

For Maximum likelihood (ML) analysis, MrModeltest 2.0 [53], a simplified version of Modeltest [54], [55], [56], was used to select the best-fitting nucleotide substitution model, after which PAUP* settings were optimized based on the data of the selected model before searching. Then, ML analyses were performed under a partitioned scheme using RAxML 7.0.3 [57] with independent GTR+I+Γ substitution models defined for each partition. The data were correspondingly partitioned into COI, tRNA/COII, CytB, 12S/16S, and EF1α. Bootstrap analysis was also performed in RAxML, with 1000 bootstrap replicates from which a majority rule consensus tree was constructed in PAUP* for identification of supported clades.

**Figure 1:**
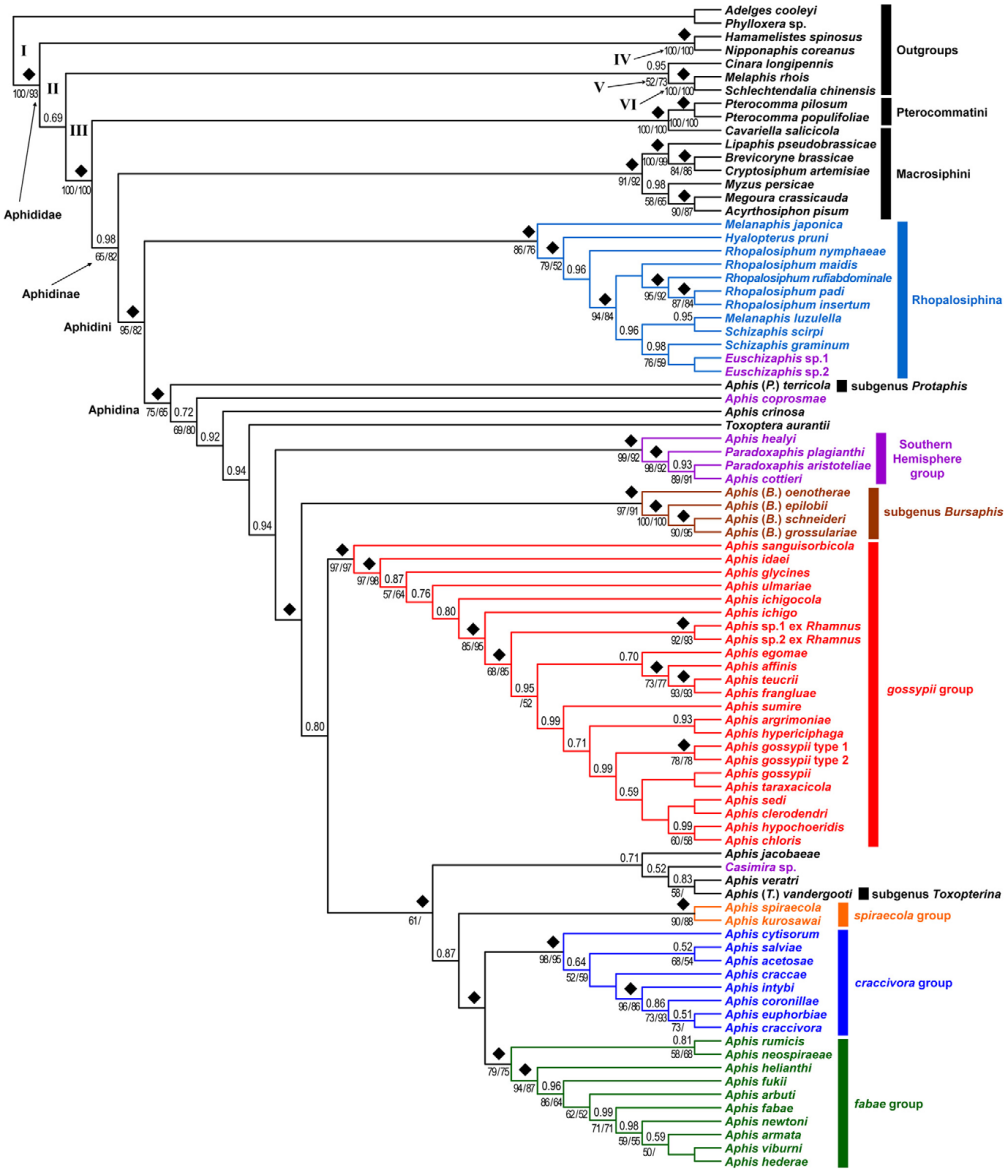
Cladogram representing the best ML topology tree of the Aphidini, Macrosiphini, and Pterocommatinae. Numbers above nodes indicate Bayesian posterior probabilities (PP), and numbers below nodes indicate ML bootstrap support values, followed by MP bootstrap support values. All support values are shown, if greater than 50%. ♦ indicates PP = 100.

Bayesian inference (BI) analyses were performed using MrBayes version 3.1.2 [58]. The best-fitting nucleotide substitution models (GTR+I+Γ) and estimated parameters for each of the five partitions were selected using the hierarchical likelihood ratio test implemented in MrModeltest. Markov-Chain Monte Carlo (MCMC) analysis was carried out with one cold and three heated chains (temperature set to 0.1; starting from a random tree). The number of generations of the MCMC analysis and the tree sampling frequency were 10 million and 100 generations, respectively. The critical value for the topological convergence diagnostic of the preliminary tests was checked with MCMC options of ‘stoprule = yes’ and ‘stopval = 0.01’. The burn-in parameter was estimated empirically by plotting −ln *L* against the number of generations using Tracer version 1.5 [59], and the trees corresponding to the first 20% generations were discarded. To ensure that the analyses were not trapped in local optima, five independent MrBayes runs were performed, after which topologies and posterior probabilities (PP) from different runs were compared for congruence purposes. We summarized the consensus tree using the post burn-in trees from all five runs in MrBayes (Fig. 1).

The effects of missing data or genes were assessed because 39 taxa among a total of 87 in this study were missing 15–68% of their sequences (Table S1). Generally, if enough characters have been sampled accurately to place all incomplete taxa on the tree, then the missing data will have little effect [60], [61], [62], [63], [64], [65]. However, if a critical topological conflict or long branch attraction arises in the phylogenetic analyses due to inclusion of the taxa missing data, then the taxa cannot be used for estimation of divergence times [63], [65], [66], [67]. To verify this, three different combined datasets were analyzed: the first one was a perfect concatenated dataset (48 taxa), the second one included taxa with at least three gene fragments (63 taxa [48 complete plus 15 missing 15–53% of their data]), and the third including all available taxa (87 taxa [63 previoiusly described plus 24 missing 67–68% of their data]). MP, ML, and BI analyses were conducted following the same methodology, after which the nodal support values of significant group clusters (e.g., subfamily, tribe, subtribe, species-group) were compared for estimation of divergence times among the analyses of the three datasets.

Significant differences between topologies resulting from the above phylogenetic analyses, as well as topologies consistent with alternative hypotheses, were tested using the likelihood-based Kishino-Hasegawa (KH) test [68] and an approximately unbiased (AU) test [69]. To perform the KH and AU tests, the first step was to reconstruct alternative tree topologies (fully-resolved) consistent with the selected hypotheses using Mesquite version 2.6 [70]. ML heuristic searches using a GTR+I+Γ model for each partition that incorporated a topological constraint were conducted by RAxML in order to produce the highest-likelihood topology that satisfied a given hypothesis. Second, PAML version 4.2b [71] was used to produce a log file (.lnf) for the log likelihoods of site-patterns of alternative trees given the concatenated dataset. The log file generated was submitted to CONSEL version 0.1i [72] to calculate the *P*-value for each alternative topology by the AU and KH tests.

### Molecular dating and calibration points

Fossil records of aphids are restricted to the Late Cretaceous to the Tertiary, and most aphid fossils have been recovered from Canadian amber dated to 75–80 million years ago (MYA) or Baltic amber dated to 35–45 MYA [15], [16], [73]. Fossils of most extant subfamilies are known from the Eocene, but only two extant groups, Aphidinae and Neophyllaphidinae, are known from the Late Cretaceous [15], [16]. Although there are few fossils of extant aphids that can be used to infer the exact time for molecular calibration, molecular dating for aphids was attempted in previous phylogenetic studies [16], [38]. As the first reasonable estimation, von Dohlen and Moran [16] suggested divergence times of representative subfamilies in Aphididae based on analysis of the partial 12S and 16S rRNA genes. This estimate is based on crucial evidence from earlier research [37], [38] that places the biogeographic isolation and divergence of the two sumac galling aphids, *Melaphis rhois* and *Schlechtendalia chinensis*, at 48–70 MYA. Moran et al. [38] previously estimated the age of the common ancestor of Aphididae to be 160–280 MYA based on the 16S rRNA sequences of the bacterial endosymbiont *Buchnera*, although later it was recalculated to be 84–99 MYA based on the common ancestor of these two melaphidines [16], [37]. In addition, it was suggested that Aphidini and Macrosiphini diverged from one another at least 50 MYA based on fossil evidence (ca. 50 MYA) and Baltic amber. Moreover, their approximate divergence was inferred to have occurred between 50–70 MYA prior based on sequence divergences of aphid endosymbiotic *Buchnera* [74]. Moran [4], [5] also suggested that aphids acquired host alternation ability between about 30–50 MYA based on fossil evidence. Recently, the divergence of Aphididae from two sister groups, Phylloxeridae and Adegidae, was inferred to have occurred between 120–150 MYA based on fossil evidence [22]. It seems valid for a molecular time estimation of Adelgidae [22], but most of the calibration points used in this estimate were obtained from earlier dating results of aphid subfamilies [16].

**Figure 2:**
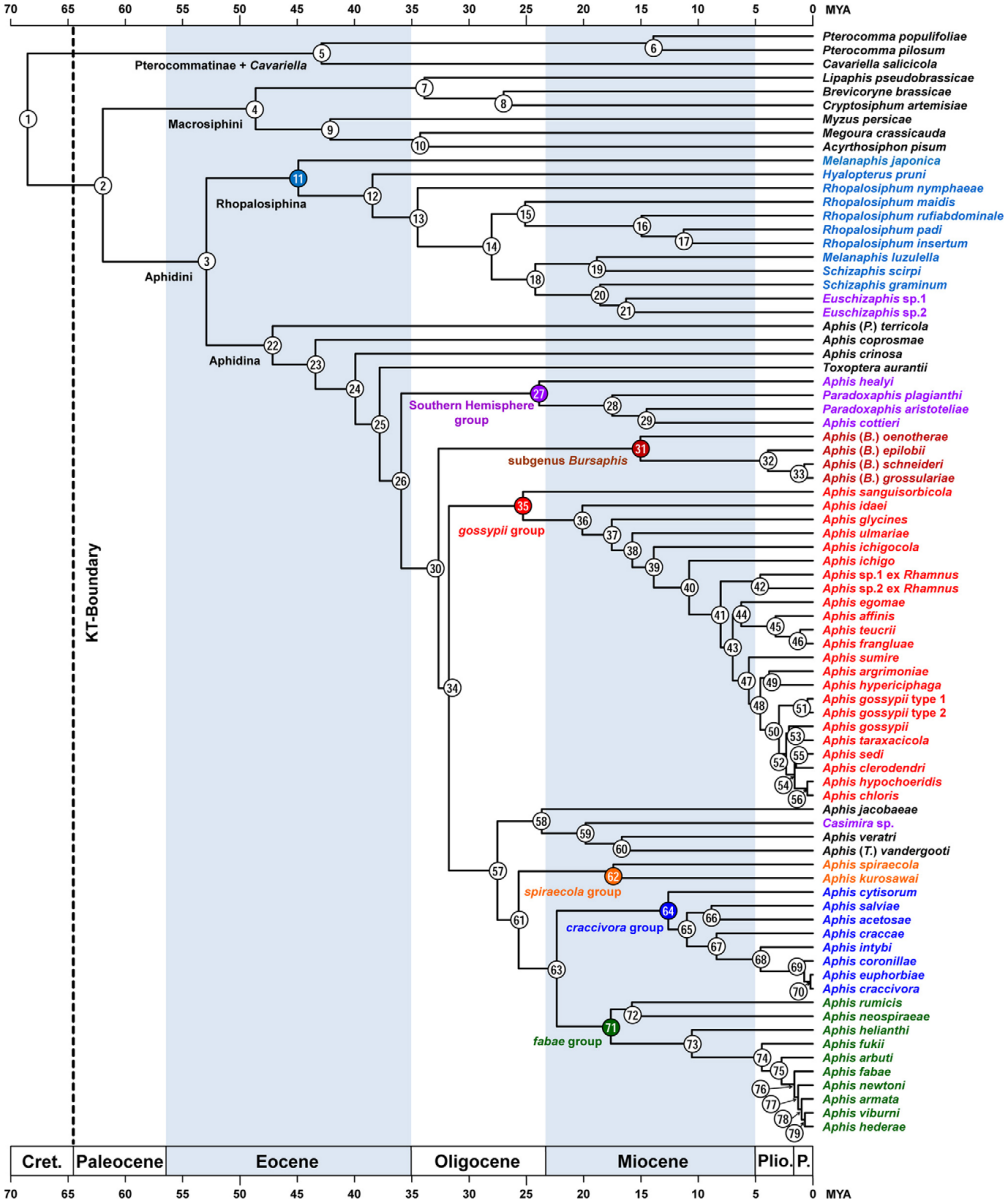
Chronogram showing the ages of origin and divergence times of the Aphidini, Macrosiphini, and Pterocommatinae. The topology corresponds to the best ML tree of Figure 1. The chronostratigraphic scale is given with absolute geological ages (MYA, million years ago; [78]). A node and species in the same color denote a clade. Numbers in circles refer to node numbers in Table 4 and Table S5. Cret. = Cretaceous. Plio. = Pliocene. P. = Pleistocene.

Therefore, based on previous studies that estimated divergence times [16], [22], calibration points required for the molecular dating analyses were assigned as follows: i) the Aphidoidea crown clade (node I in Figure 1) was fixed at 150 MYA; ii) the Aphididae crown clade was constrained at a minimum age of 80 MYA and a maximum age of 100 MYA. However, since two nodes appeared in the phylogenetic analyses (nodes II and III in Figure 1), the same age constraint was applied for both nodes; iii) the divergence point of *M. rhois* and *S. chinensis* (node V in Figure 1) was constrained at a minimum age of 48 MYA and a maximum age of 70 MYA; iv) the divergence point of Aphidini and Macrosiphini (node 2 in Figure 2) was constrained at a minimum age of 50 MYA (Appendix 2). To reduce the uncertainties of the time estimation, two Bayesian inference-based programs, MULTIDIVTIME version 09.25.03 [75], [76] and BEAST version 1.5.3 [77], were used to perform the molecular dating analyses. The geological time scale referenced is that of Gradstein and Ogg [78].

### MULTIDIVTIME analysis

PAML/MULTIDIVTIME were used following the method of Rutschmann [79]. Although some taxa were missing from the individual gene datasets, except for tRNA/COII (see Table S1), two package programs, ESTBRANCHES and MULTIDIVTIME, were able to account for the missing taxa [76]. To estimate the divergence times, a fully resolved topology of the combined dataset was obtained using RAxML (Figure 1), and this was also the best likelihood topology based on the KH and AU tests (see Results). At first, the BASEML program of PAML [71] was used to analyze the total molecular sequence data and parameters of the substitution model using the F84 model [68], [80] for each gene separately based on individually optimized topologies. PAML2MODELINF was run to convert the BASEML output to useable data for ESTBRANCHES, which was then used to estimate branch lengths and their associated variance-covariance matrix using each output file from previous analyses. In this instance, the fully resolved target tree including the missing taxa was used. The outgroups were then pruned from the tree. The mean of the prior distribution of time from the ingroup root to the tip (rttm) was set to 0.9, and its standard deviation (rttmsd) was set to 0.1, in which one time unit represents 100 million years. Following the program manual recommendations, additional priors specified were rtrate = 0.35; rtratesd = 0.35; brownmean = 1.1; brownsd = 1.1; and bigtime = 100.0. The four nodes were constrained as follows: the Aphididae crown clade (ingroup root) and the clade of [Lachninae+Eriosomatinae]+Aphidinae were equally constrained at 80–100 MYA (L = 0.8, U = 1.0); the divergence point of *M. rhois* and *S. chinensis* at 48–70 MYA (L = 0.48, U = 0.7); the divergence point of Aphidini and Macrosiphini at a minimum age of 50 MYA (L = 0.5). Even though the most basal node did not require an additional constraint in MULTIDIVTIME, the constraint was maintained in order to compare its estimated time with that from BEAST, which requires constraining the same node. Other settings were left unchanged. The MCMC algorithm completed 300,000 initial burn-in cycles before the state of the Markov chain was sampled. Thereafter, the Markov chain was sampled every 100th generation until a total of 30,000 samples were collected. To test whether or not the Markov chain was convergent, three independent replicates were carried out.

### BEAST analysis

A second analysis was performed using the BEAST software package 1.5.3 [77], which is designed to estimate divergence times using a Bayesian MCMC approach. At first, the software tool BEAUti 1.5.3 of the BEAST package [77] was employed to design the run-file for BEAST. The uncorrelated lognormal model was used to describe the relaxed-clock, whereas GTR+I+Γ was used to describe the substitution model. A Yule prior was used on the tree to simulate the process of speciation. In the BEAST analyses, the Aphidoidea crown ([Adelgidae+Phylloxeridae]+Aphididae) was fixed at 150 MYA, and the three other nodes were constrained according to the settings of the previous MULTIDIVTIME analyses. A preliminary test of MCMC run with 10 million generations was first performed to optimize the scale factors of the priori function. The final MCMC chain was run twice for 100 million generations sampled every 1000th generation. A 10% burn-in was discarded from the beginning of each run, and all samples were examined using Tracer 1.5 [59] to verify convergence and an effective sample size exceeding at least 200 for all parameters estimated. TreeAnnotater 1.5.3 of BEAST package [77] was used to summarize the mean parameter estimates and 95% highest posterior densities (HPDs), and then FigTree 1.3.1 [81] was used to visualize the results, including the confidence intervals.

### Ancestral state reconstruction

Two ancestral states of the Aphidini, biogeography and host alternation, were reconstructed according to a Bayesian criterion [82] using BayesMultiState implemented in BayesTraits version 1.0 [83]. This method can allow for both polymorphism of character states and uncertainty in phylogeny. To reduce the uncertainty and arbitrary nature of choosing priors under MCMC, the reverse jump hyperprior approach (the rjhp command) was used as recommended [82], [83]. For each test, combinations of hyperprior values (exponential or gamma, mean and variance) and rate parameter values were explored in order to find acceptance rates when running Markov chains between 20 and 40% (as recommended by [83]). A reverse jump hyperprior exponential (rjhp exp 0.0 30) distribution with a rate deviation prior of 10 was employed to analyze area, and a rjhp exp 0.0 2 with a rate deviation of 50 was used in the analysis of host alternation. Since tree branch length was important in this analysis, the 10 ML topology trees which showed similar best likelihood scores in the RAxML analyses were explored. The MCMC chain was run twice for 100 million generations sampled every 1000th generation after a burn-in of 10 million generations. The stationary phase during the MCMC run was observed by plotting the harmonic mean and then looking for a plateau, after which the means of each prior were calculated.

To reconstruct the ancestral state of host alternation, three states were identified: (0) monoecious holocyclic (mon. hol.) on an herbaceous plant, (1) mon. hol. on a shrubby or woody plant, (2) host alternation (heteroecy). Detailed information for coding the character state is given in Table S3. In this analysis, one anholocyclic species, *Toxoptera aurantii*, was regarded as holocyclic, whereas some species varying facultatively or genetically between anholycyclic and heteroecious (e.g., *Aphis gossypii*) were designated as host-alternating. Macrosiphini and Pterocommatinae were not included upon inferring two ancestral reconstructions for Aphidini.

For reconstruction of the ancestral state of area, the possible origin of the distribution of each species was coded into four regions based on the previous distribution records (e.g., Stroyan [84], Heie [85], Blackman and Eastop [14], Teulon and Stufkens [86], and Lee et al. [87]): (A) European (with some regions in the Western Palearcic), (B) Asian (with some regions in the Eastern Palearctic), (C) Australasian, and (D) Neartic. Detailed information for coding the character states is given in Table S4. Because several taxa occurring in more than one region could not be coded to one state, the multiple character state option was used, which can be assigned in BayesMultistate: Palearctic (AB), Palearctic+Nearctic (ABD), Cosmopolitan (ABCD). According to the BayesTraits manual [83], the code AB signifies that a trait can be in states A or B (with equal probability) but not in states C or D. Tropical areas, i.e., Afrotropical, Indo-Malayan, and Neotropical regions, were excluded since aphids are thought to have originated in temperate regions, especially the Northern Hemisphere [21], [25].

## Results

### Phylogenetic analysis

In the comparison of individual gene datasets, CytB had the largest proportion of informative characters (32.2%) as well as the greatest pairwise sequence divergence (8.0%) between ingroup species among the five DNA regions (Table 1). In contrast, the nuclear EF1α had the smallest sequence divergence among all sequence regions, and the sequence divergence of 12S/16S was the lowest among all mitochondrial regions. Regarding the Ti/Tv ratio, three mitochondrial genes showed moderate ratios (ca. 1.25), whereas 12S/16S showed predominance of Tv (0.389). On the contrary, EF1α showed a predominance of Ti (2.333). The partition-homogeneity test [52] showed no significant evidence of phylogenetic conflicts between the two paired regions or within the combined dataset (0.07≤*P*≤0.91). Thus, these five regions are expected to account for different taxonomic levels, suitable for this phylogenetic reconstruction.

**Table 1:** Characteristics of DNA sequences and three combined datasets.

	Single individual datasets					Combined datasets		
	COI	tRNA/COII	CytB	12S/16S	EF1α	CDS-48	CDS-63	CDS-87
Number of taxa	60	87	76	51	62	48	63	87
Aligned sequence length (bp)	658	702	737	1601	802	4500	4500	4500
Variable sites (%)	247 (37.5)	310 (44.2)	325 (44.1)	600 (37.4)	230 (28.7)	1568 (34.8)	1682 (37.4)	1712 (38.0)
Informative characters (%)	208 (31.6)	224 (31.9)	237 (32.2)	324 (20.2)	180 (22.4)	1046 (23.2)	1156 (25.7)	1173 (26.1)
Nucleotide composition (T∶C∶A∶G)	41∶14∶35∶10	39∶12∶41∶8	43∶13∶35∶9	46∶5∶38∶11	26∶22∶28∶24	40∶12∶36∶12	40∶12∶36∶12	40∶12∶36∶12
Pairwise sequence divergence*	7.5±2.2	6.0±2.2	8.0±2.8	3.8±1.5	3.6±2.3	5.3±1.9	6.2±2.2	6.0±2.1
Ti/Tv ratio	1.216	1.281	1.250	0.389	2.333	0.969	1.135	1.250

To reconstruct the phylogeny of Aphidini, we used one nuclear and four mitochondrial DNA sequences. Due to missing sequence data, we compared three different combined datasets.

*indicates uncorrected *P*-distance (mean ± S.D.) among ingroup species.

The effects of the missing data or genes (15–68%) were assessed using MP, ML, and BI analyses with the three different combined datasets (Table 2). Hereafter, the combined datasets (CDS) with 48, 63, and 87 taxa are abbreviated to CDS-48, CDS-63, and CDS-87, respectively. For each CDS, the best-fitting model of nucleotide substitution was GTR+I+Γ in both the ML and BI analyses. No topological conflict was identified among the three datasets, as the most important nodes in each dataset were recovered. The statistical support values of the datasets were compared with the 17 important nodes responsible for the subfamily, tribal, subtribal, and species-group clusters. Bootstrap values estimated in the MP and ML analyses were significantly affected by inclusion of the taxa with missing data, whereas posterior probabilities of the BI analysis were relatively less sensitive. However, the ML or BI support values between CDS-63 and CDS-87 increased on several nodes upon inclusion of the taxa with missing data (Table 2). This implies that the taxa with missing data could be used to corroborate each clade without topological conflict. In the molecular dating analysis, the topologies in the BI analysis were chosen rather than those in the ML or MP analysis, since both BEAST and MULTIDIVTIME estimated the divergence times under the Bayesian algorithm-based clock model [76], [77]. Because there seemed to be no significant difference in support values of the BI analysis among three datasets, CDS-87 was used for both phylogenetic reconstruction and estimation of divergence times.

**Table 2:** Statistics of support values estimated from three combined datasets.

		87 taxa			63 taxa			48 taxa		
Node no.	Node description	BI	ML	MP	BI	ML	MP	BI	ML	MP
1	Aphidinae+Pterocommatinae	1.00	100	100	1.00	100	100	1.00	100	100
2	Macrosiphini+Aphidini	0.98	65	82	0.98	63	83	1.00	69	69
3	Aphidini	1.00	95	82	1.00	98	89	1.00	96	77
4	Macrosiphini	1.00	91	92	1.00	91	98	1.00	87	94
5	Pterocommatinae	1.00	100	100	1.00	100	100	1.00	100	100
11	Rhopalosiphina	1.00	86	76	1.00	84	80	1.00	82	65
12	clade sister to *M. japonica*	1.00	79	52	1.00	80	53	1.00	77	58
22	Aphidina	1.00	75	65	1.00	100	97	1.00	100	99
24	clade sister to *A. crinosa*	0.94	37	20	0.83	43	43	1.00	65	68
25	clade sister to *T. aurantii*	0.94	24	24	0.76	23	28	1.00	92	68
30	clade sister to Southern Hemisphere group	1.00	39	23	0.86	48	31	-	-	-
34	clade of four species groups with other spp.	0.80	29	15	0.86	35	18	1.00	41	-
35	*gossypii* group	1.00	97	97	1.00	100	99	1.00	100	100
61	*craccivora*+*fabae*+*spiraecola* groups	0.87	36	26	1.00	84	62	1.00	92	77
63	*craccivora*+*fabae* groups	1.00	42	35	1.00	50	55	0.51	43	48
64	*craccivora* group	1.00	98	95	1.00	100	100	-	-	-
71	*fabae* group	1.00	79	75	1.00	96	96	1.00	97	99

We used BI, ML, and MP analyses to compare the datasets. Node no. refers to nodes of phylogeny in Figure 2.

**Figure 3:**
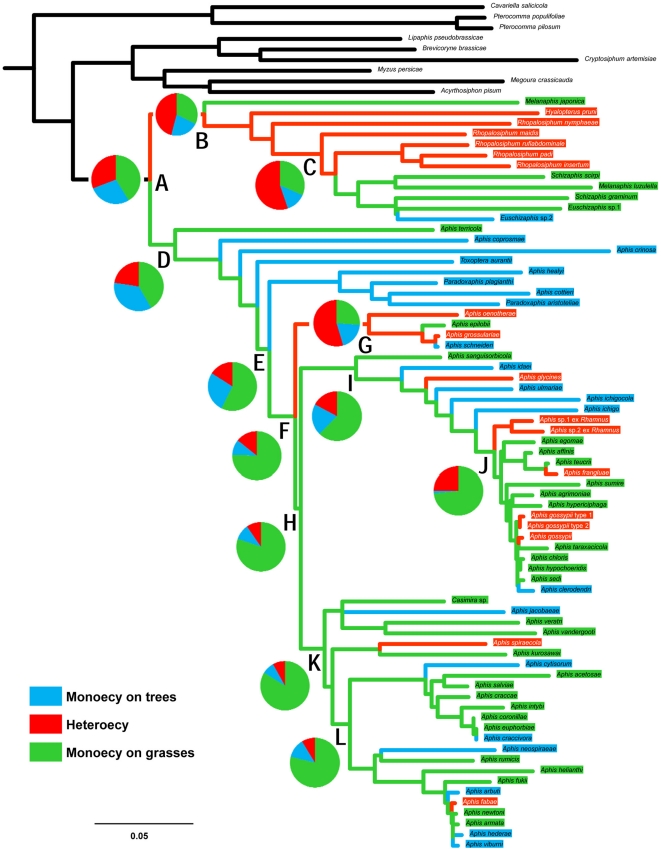
Ancestral state reconstruction for host alternation. The ancestral states are classified into monoecy on trees (blue), heteroecy (red), and monoecy on grasses (green). The topology is derived from the ML tree of Figure 1. Pie charts indicate the relative likelihoods at respective nodes (A–L). Terminal taxa and their respective branches are color-coded for state of host use. The scale is a nucleotide substitution rate of 0.05.

**Figure 4:**
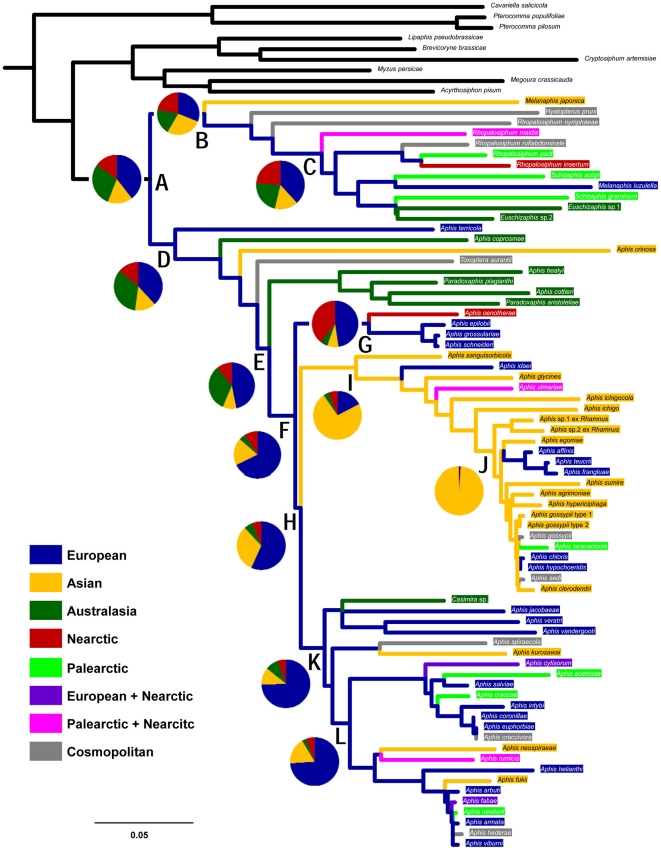
Ancestral state reconstruction for biogeographic origin. The topology is derived from the ML tree of Figure 1. Pie charts indicate the relative likelihoods at respective nodes (A–L). Terminal taxa and their respective branches are color-coded for state of host use. The scale is a nucleotide substitution rate of 0.05. Palearctic, European+Nearctic, Palearctic+Nearctic, and cosmopolitan states were coded as multistate and thus do not appear in pie charts.

The ML tree based on the best likelihood score corresponds to the 50% majority rule consensus tree of the BI analysis, except for some unresolved clades (Figure 1). Due to large genetic distances between the outgroup and ingroup species, the cladogram is illustrated showing only relationships instead of the phylogram. The relative genetic distances between ingroup species can be seen in Figures 3 and 4. The clade consisting of Aphidinae+Pterocommatinae (node 2 in Figure 2) was well supported in all analyses. In this study, the P-C group was the most basal tribe within Aphidinae, but it was not robustly supported with 0.98 PP or the 65% ML bootstrap value. Except for the P-C group, all other tribal and subtribal clades received 1.0 PP and a ML-bootstrap value ranging from 75 to 95%. The tribe Macrosiphini was separated from the tribe Aphidini,which in turn was subdivided into two monophyletic subtribes, Aphidina and Rhopalosiphina. Within Rhopalosiphina, *Melanaphis japonica* was sister to the remaining rhopalosiphine species with 1.0 PP and a 79% ML bootstrap value. In the BI analysis, *Melanaphis luzullella* was not clustered with *M. japonica* but was closely related with *Schizaphis* species. Within Aphidina, *Aphis terricola*, *A. coprosmae*, and *A. crinosa* appeared sequentially in the basal nodes. These three species are suggested to be the most basal taxa of all Aphidina species, even though the sister clade of *A. crinosa* received low support values (0.72–0.94 PP). Although these three species did not form a clade, *A. crinosa* and *A. coprosmae* were most likely transferred to the subgenus *Protaphi*, because their morphological characters were consistent with those of *Protaphis* [85], [86], [88]. As the sister group of the node of *Toxoptera aurantii*, four Southern Hemisphere species clustered as a sister group consisting of the remaining *Aphis* species, which were robustly supported in the BI analysis (1.0 PP). Except for the genus *Bursaphis*, most *Aphis* species were partitioned into two subclades, the *gossypii* group and the *craccivora*+*fabae*+*spiraecola* groups. Each of these four species groups was highly supported by 1.0 PP and a ML bootstrap value ranging from 79 to 98%.

The results of the KH and AU tests of the alternative tree topologies are summarized in Table 3. The two alternative sister relationships with Pterocommatinae were not significantly different, but their confidence values (0.086–0.171) were too low to replace the best topology. Of ten alternative tree topologies tested with respect to the basal position within Aphidina, seven were rejected (*P*<0.05). In particular, the basal location of all six Southern Hemisphere species within Aphidina was accepted even with low confidence values. For the tests of the basal position within Rhopalosiphina, only the alternative position of the genus *Hyalopterus* was allowed with narrow confidence values. Three possible monophylies were tested, and then the monophyly of the genus *Rhopalosiphum* was rejected. Consequently, although eight alternative topologies were accepted (Table 3), they received much lower confidence values, ranging from 0.062 to 0.253, than did the best topology.

**Table 3:** Comparison between the best (1) and the alternative (2–20) topologies.

Topology	Description of alternative topology	Rank	Obs	KH	AU
1	Best ML tree from RAxML	1	----	0.522	0.823
2	Secondly best ML tree from RAxML	2	0.3	0.478	0.709
3	(Pterocommatinae+Aphidini)+Macrosiphini	7	17.8	0.086	0.129
4	(Pterocommatinae+Macrosiphini)+Aphidini	4	14.7	0.089	0.171
5	Basal position of *Bursaphis* within Aphidina	16	46.3	0.019*	0.024*
6	Basal position of *gossypii* group within Aphidina	18	54.3	0.022*	0.021*
7	Basal position of (*craccivora*+*fabae*+*spiraecola* groups)+node 58 within Aphidina	15	44	0.019*	0.022*
8	Basal position of node 30 within Aphidina	13	31.8	0.023*	0.014*
9	Basal position of *craccivora*+*fabae*+*spiraecola* groups within Aphidina	17	52.9	0.01*	0.016*
10	Basal position of all Southern Hemisphere species (non-monophyly) within Aphidina	14	39.4	0.022*	0.019*
11	Basal position of *A. coprosmae*+Southern Hemisphere group within Aphidina	11	24	0.076	0.157
12	Basal position of *A. coprosmae* within Aphidina	3	11.2	0.09	0.253
13	Basal position of *A. crinosa* within Aphidina	6	17.6	0.025*	0.059
14	Basal position of *T. aurantii* within Aphidina	12	25.2	0.067	0.108
15	Monophyly of four species groups excluding node 58	8	22.3	0.062	0.092
16	Monophyly of *Rhopalosiphum*	10	23.8	0.031*	0.04*
17	Monophyly of *Melanaphis*	9	22.3	0.065	0.086
18	Basal position of *Hyalopterus* within Rhopalosiphina	5	15.5	0.081	0.104
19	Basal position of *Rhopalosiphum* (non-monophyly) within Rhopalosiphina	19	64.8	0.001*	<0.001*
20	Basal position of (*Schizaphis*+*Euschizaphis* [non-monophyly]) withiin Rhopalosiphina	20	65.2	0.007*	0.006*

Nodes 30 and 58 are those referred in Figure 2.

*signifies that the hypothesis received a *P* value<0.05 and can be rejected.

### Divergence times

The estimated divergence times for the 33 selected nodes of the chronogram (Figure 2) are summarized in Table 4, and those for all nodes are shown in Table S5. Mean age estimates of the 33 nodes were slightly different, averaging 1.82 MYA between the MULTIDIVTIME and BEAST analyses. However, 95% HPDs of the BEAST analyses generally overlapped with those of the MULTIDIVTIME analyses, suggesting that the time estimates of the two programs were largely congruent. Based on the results of the MULTIDIVTIME and BEAST analyses, the divergence point of the P-C group and Aphidinae was estimated to be immediately before the K-T boundary (67–68 MYA), whereas the divergence of Aphidini and Macrosiphini occurred after that (62 MYA). The divergences within the tribal and subtribal clades arose in the Early to Middle Eocene (42–55 MYA). Within the Pterocommatinae+Cavariella group, the divergence between Pterocamma and Cavariella was dated to ca. 42 MYA. The divergences of the rhopalosiphine genera occurred over a considerable interval. That is, Melanaphis diverged first near the Middle Eocene (45–50 MYA), whereas Schizaphis emerged during the Late Oligocene (24–30 MYA). Within Aphidina, the divergence times of the most extant members in the subgenus or species-group were estimated to be in the Late Oligocene to Middle Miocene (12–25 MYA). Some morphologically cryptic species in gossypii- and fabae-groups arose mostly after the Pliocene (<5 MYA). In summary, most generic divergences in Aphidini occurred in the Middle Tertiary, and species-level divergences occurred in the Middle or Late Tertiary.

**Table 4:** Estimated divergence times for selected nodes.

		BEAST		Multidivtime	
Node no.	Node explain	Time	95% HDP range	Time	95% HDP range
II	Aphididae	97.0	(91.5–100.0)	94.2	(85.1–99.7)
III	(Lachninae+Eriosomatinae)+ingroup clade	94.2	(87.9–99.3)	92.4	(83.5–98.7)
IV	Hormaphidinae	65.7	(48.5–82.5)	70.8	(57.5–84.0)
V	Lachninae+Eriosomatinae	83.7	(73.9–93.4)	87.7	(77.3–96.4)
VI	Eriosomatinae	52.4	(48.0–59.6)	51.5	(48.1–59.4)
1	(Pterocommatinae+*Cavariella*)+Aphidinae	68.5	(58.8–78.5)	67.2	(58.7–77.4)
2	Macrosiphini+Aphidini	62.0	(52.9–71.0)	62.2	(54.5–71.9)
3	Aphidini	52.9	(45.0–61.4)	55.0	(50.2–63.9)
4	Macrosiphini	48.6	(38.4–58.9)	48.9	(39.9–59.3)
5	Pterocommatinae+*Cavariella*	42.9	(29.6–55.9)	42.2	(31.9–53.8)
11	Rhopalosiphina	44.9	(36.9–53.2)	50.6	(43.8–59.7)
12	divergence of *H. pruni*	38.4	(31.2–46.4)	42.0	(34.5–51.2)
14	*Rhoplosiphum*+*Schizaphis* clade	28.0	(22.2–34.6)	33.0	(25.7–41.8)
18	*Schizaphis* (including *M. luzulella*)	24.2	(18.5–30.4)	29.7	(22.3–38.3)
22	Aphidina	47.2	(38.6–55.6)	50.4	(42.6–60.0)
23	divergence of *A. coporosmae*	43.4	(36.0–51.1)	45.6	(38.4–54.6)
24	divergence of *A. crinosa*	39.9	(32.9–47.2)	43.1	(36.1–52.0)
25	divergence of *T. aurantii*	37.8	(31.4–44.6)	39.1	(32.0–47.8)
26	divergence of Southern Hemisphere group	35.9	(29.6–42.5)	35.7	(28.5–44.4)
27	clade of Southern Hemisphere group	23.9	(16.9–30.8)	24.8	(16.6–34.5)
30	clade sister to Southern Hemisphere group	32.7	(27.1–38.7)	30.4	(23.8–38.7)
31	clade of subgenus *Bursaphis*	15.0	(8.2–22.8)	13.6	(6.4–22.4)
34	clade of four species groups with other spp.	31.8	(26.3–37.6)	28.0	(21.7–35.9)
35	*gossypii* group	25.3	(20.2–30.6)	20.4	(14.7–27.6)
37	divergence of *A. glycines*	17.5	(13.5–21.8)	15.0	(10.5–20.9)
41	divergence of two *Aphis* spp. ex *Rhamnus*	8.0	(6.0–10.1)	6.4	(3.9–9.8)
48	morphologically cryptic species with *A. gossypii*	4.6	(3.1–6.1)	2.9	(1.5–4.9)
61	*craccivora*+*fabae*+*spiraecola* groups	25.7	(20.8–30.8)	23.1	(17.3–30.3)
62	*spiraecola* group	17.4	(11.4–23.3)	17.1	(11.4–23.9)
63	*craccivora*+*fabae* groups	22.4	(17.6–27.2)	19.8	(14.3–26.7)
64	*craccivora* group	12.6	(8.7–16.9)	11.7	(6.2–19.0)
71	*fabae* group	17.6	(13.2–22.3)	16.4	(11.6–22.4)
74	morphologically cryptic species with *A. fabae*	4.5	(2.5–6.6)	3.6	(1.8–6.1)

Node numbers refer to those in Figures 1 and 2. Roman numerals represent outgroups.

### Evolution of host plant association and host alternation

The estimation of divergence times suggests that Pterocommatinae and Aphidinae likely diversified during the radiation period of their host plants (Tables 4, 5). The divergence between Aphidinae and the P-C group (node 1 in Figure 2) likely occurred along with early diversification of Rosaceae [89], [90]. The divergence times of *Pterocomma* and *Cavariella* (node 5) were inferred to be near the earliest fossil record of Salicaceae [11] and Araliaceae [89]. The divergence between Aphidini and Macrosiphini (node 2) in the Middle Paleocene overlapped the periods suggested by the earliest fossil record of Rosaceae [91] and by the molecular dating results for Rosaceae [89]. In addition, four basal divergences for Macrosiphini, Aphidini, Aphidina, and Rhopalosiphina (nodes 3, 4, 11, 22) within Aphidinae were embedded within the initial divergence periods of Rosaceae [89], [91].

**Table 5:** Divergence times or earliest fossil occurrences of host plants for Aphidinae and Pterocommatinae aphids.

Host-plant taxon	Epoch	Age (MYA)^a^	Method^b^	Related aphid taxon (node no.)	References
*Artemisia*	Middle to Late Miocene	10–23^c^	Fossil	*Aphis kurosawai* (62)	[112]
Araliaceae	Middle Eocene	41–44	Dating (N)	P-C group (5)	[89]
Asteraceae	Early to Middle Eocene	51	Dating (N)	*Aphis terricola* (22)	[92]
Asteraceae	Early Eocene	42–48	Dating (P)	*Aphis terricola* (22)	[93]
*Centaurea* (Cardueae)^d^	Late Eocene	35–38	Dating (N)	*Aphis terricola* (22)	[92]
*Coprosma* (Anthospermeae)^d^	Middle Oligocene to Middle Eocene	31.8–47.6	Dating (B)	*Aphis coprosmae* (23)	[94]
*Epilobium* e and *Oenothera*	Oligocene to Miocene	12–35	Dating (P)	*Bursaphis* (31)	[99], [100]
*Glycine*	Early to Middle Miocene	14.2–19.2	Dating (P)	*Aphis glycines* (37)	[97]
*Glycine*	Early to Middle Miocene	8.0–11.0	Dating (P)	*Aphis glycines* (37)	[98]
*Ligustrum* (Oleaceae)^d^	Middle Paleocene to Late Eocene	37–64	Dating (N)	*Aphis crinosa* (24)	[89]
*Miscanthus* (Paniceae)^d^	Early Miocene	20.6	Dating (M)	*Melanaphis japonica* (11)	[101]
*Phragmites*	Early Miocene	17.8–20.6	Dating (M)	*Hyalopterus pruni* (12)	[101]
*Prunus*	Middle Eocene	48^f^	Fossil	*Hyalopterus pruni* (12)	[11]
*Prunus*	Middle Eocene	35	Dating (N)	*Hyalopterus pruni* (12)	[89]
*Rhamnus* (including *Frangulae*)	Late Oligocene	26.5–27.4	Dating (N)	*gossypii* group (35)	[95]
*Ribes* (Saxifragaceae)^d^	Late Cretaceous	89–96	Dating (P)	*Bursaphis* (31)	[96]
*Ribes* (Saxifragaceae)^d^	Late Cretaceous	81	Dating (N)	*Bursaphis* (31)	[89]
Rosaceae	Middle Eocene	35–56^c^	Fossil	Aphidini+Macrosiphini (2, 3, 4)	[91]
Rosaceae	Late Cretaceous to Middle Eocene	44–76	Dating (N)	Aphidini+Macrosiphini (2, 3, 4)	[89]
Salicaceae	Middle Eocene	45^f^	Fossil	P-C group (5)	[11]
*Salix* and *Populus*	Paleocene	60–65	Fossil	Aphidinae+P-C group (1)	[90]
Spiraeoideae ( = Prunoideae)	Middle Miocene to Early Oligocene	29–44	Dating (N)	*Hyalopterus pruni* (12)	[89]

a : geological time scale from Gradstein and Ogg [78].

b : fossil means the earliest fossil record. Dating method in parenthesis: B, relaxed-clock in BEAST; M, relaxed-clock in MULTIDIVTIME; N, non-parametric rate smoothing in R8S; P, penalized likelihood in R8S.

c : range of the epoch period.

d : age inferred from the higher taxon.

e : inferred by phylogenetic relationships with *Fuchsia* and *Oenothera*.

f : absolute age based on the earliest fossil by Lopez-Vaamonde et al. [11].

Three *Protaphis*-like species, *Aphis terricola*, *A. coporosmae*, and *A. crinosa*, placed basally within Aphidina, diverged during the Eocene and corresponded to the appearances of their host plants, Asteraceae, Rubiaceae, and Oleaceae, respectively [92], [93], [94]. Divergences of the *Melanaphis* (node 11) at 45–50 MYA and the *Hyalopterus* (node 12) at 38–43 MYA were also similar to the appearances of *Prunus* or Spiraeoideae [11], [89]. The divergence point of the most recent common ancestor (MRCA) of the *gossypii* group was estimated at 20–25 MYA and overlapped the divergence times of its primary hosts, *Rhamnus* and *Frangula* [95], assuming that the MRCA of the *gossypii* group (node 35) associated with these hosts. Therefore, the divergence times between generic or subgeneric level taxa of aphids and their primary hosts are almost consistent. However, *Ribes*, a primary host genus of the subgenus *Bursaphis* that diverged in the Miocene, likely diversified in the Late Cretaceous [96].

In comparison of the divergence times between aphid taxa and their secondary hosts, one species of the *gossypii* group, *Aphis glycines* (node 37), was estimated to have diverged at 15–17 MYA, which was precisely nested within the estimated times of *Glycines*, its secondary host species [97], [98]. The divergence times of *Epilobium* and *Oenothera* [99], [100] are closer to those of *Bursaphis* species (nodes 31). However, the estimated divergence times of *Hyalopterus* differ considerably from those of *Phragmites* (17–20 MYA) [101], which is the sole secondary host genus of *Hyalopterus* [14]. Similarly, the divergence of *Melanaphis* (node 11) is much earlier than that of the host *Miscanthus* [101]. Therefore, the divergence times of secondary hosts are more consistent with those of aphid species than those of genera.

No ancestral state for Aphidini (node A in Figure 3) was highly favored from among the three states: monoecy on trees, heteroecy, and monoecy on grasses. However, the two monoecious states were combined, monoecy had a higher probability than did heteroecy. Two nodes, B and C, in Rhopalosiphina showed near half proportions of heteroecy, 0.46 and 0.55, respectively, with regards to the origin of host alternation. *Melanaphis* aphids exhibit both heteroecy and monoecy on grasses. When the ancestral state of *Melanaphis japonica* was set to heteroecy, the proportion of the host alternation at node B increased to 0.51. However, the ancestral state of Aphidina (node D) was more likely monoecy on grasses (0.42) or monoecy on trees (0.36) than heteroecy (0.23). In general, ancestral host alternation was inferred to be less likely within Aphidina (nodes E, F, and H-L; 0.08–0.25), except for the clade of *Buraphis* (node G; 0.55). In addition, more recent groups (nodes H-L) within *Aphis* were highly inferred (0.76–0.84) to have originated from an ancestor that was monoecious holocyclic on herbaceous plants. Thus, the ancestral state of Rhopalosiphina seemed to be equivocal between heteroecy and monoecy, whereas that of Aphidina seemed to be monoecy.

### Biogeographic origins

The origin of Aphidini was not clearly inferred to one region; both the European and Australasian regions received relatively high probabilities of 0.38 and 0.29, respectively (node A in Figure 4). Within Rhopalosiphina, the exact distributional origins also could not be predicted at nodes B and C, but the European region had the highest probabilities of 0.31 and 0.38, respectively, among all regions. The probability of an Australasian origin for Aphidina (node D) was 0.34, probably due to the basality of certain Southern Hemisphere species, although the European origin still constituted the largest proportion at 0.38. Thus, Aphidina probably diverged into European and Australasian lineages early in its evolution. Subsequent to that, large proportions of European ancestral origin were highly inferred for both nodes F and H, which radiated to the Nearctic region (node G) and subsequently to the Asian region (node I) at 28–33 MYA. Correspondingly, most Asian species originated from the MRCA of the *gossypii* group (nodes I and J), whereas the *craccivora*, *fabae*, and *spiraecola* groups more likely originated from the European ancestor (nodes K and L). Based on these results, morphological separation between the species-groups and morphological stasis within each species-group [27] may be caused by the regional isolation of the two conspicuous lineages that originated in the European and Asian regions.

## Discussion

### Phylogenetic relationships of Aphidini, Macrosiphini, and Pterocommatinae

The phylogeny presented in this study shows that the P-C group containing two genera, *Pterocomma* and *Cavariella*, is the most basal group of Aphidinae (Figure 1). Indeed, *Cavariella* should be transferred into Pterocommaninae, because these groups share two common features: 1) primary host association with Salicaceae [2], [14] and 2) morphological characteristics of fundatrices that are almost identical to their offspring [102], [103]. Our phylogeny is consistent with phylogenies based on morphological characters, retaining the independent subfamiliy of Pterocommatinae [24], [84], [85]. The Pterocommatinae diverged early from the Aphidinae, and then Aphidinae diverged into Macrosiphini and Aphidini more recently.

In an earlier study based on a combination of two gene regions, tRNA/COII and EF1α, von Dohlen et al. [21] suggested that the P-C group had a sister group relationship with Macrosiphini. Although the KH and AU tests in this study did not reject the two alternative topologies, i) ([P-C group+Aphidini]+Macrosiphini) and ii) ([P-C group+Macrosiphini]+Aphidini), the confidence values of both tests were approximately one-fifth of the best topology of (P-C group+[Aphidini+Macrosiphini]). Furthermore, the P-C group had relatively large genetic distances from Aphidini and Macrosiphini and also exhibited a long-branch from the root in both the ML and BI analyses. Our phylogeny is also consistent with the recent phylogeny by Ortiz-Rivas and Martinez Torres [36], in which two nuclear genes, long-wave length opsin and ATP6 (1,360 bp), were used together with tRNA/COII and EF1α. Our study based on an alternative combination of mitochondrial and nuclear sequences also supports the basality of the P-C group.

The monophyly of both Aphidina and Rhopalosiphina was well supported and corresponded to previous phylogenies of Aphidini [25], [26]. Although five DNA regions (4,500 bp) were used for the analyses in this study, the monophyly of *Rhopalosiphum*, *Melanaphis*, and *Schizaphis* was not resolved. Moreover, two *Melanaphis* species did not form a clade as they adapted to two unrelated plant genera, *Micanthus* and *Luzula* [14]. In *Aphis*, however, each of four species-groups and the subgenus *Buraphis* clearly formed monophyly, even though the Aphidina species were genetically closer to one another than the Rhopalosiphina species. The inconsistencies between the taxonomic and phylogenetic relationships are likely caused by faulty diagnoses for the genera of Rhopalosiphina [84], [85]. Thus, the generic division and classification within Rhopalosiphina need to be revised.

### Evolution of host plant association and host alternation

Although molecular dating remains controversial due to different molecular rates across lineages [104], this technique is widely used for phylogenetic reconstruction and determining evolutionary patterns [10], [11]. The estimation of divergence times suggests that aphid taxa used in this study likely diversified during the radiation period of their host plants (Table 4, 5). The diversification periods of aphid taxa and their hosts were overlapping, even though the divergence time estimates for hosts differed depending on which dating methods and fossil information were used (Figure 2; Table 5). However, the time estimates could not give an explanation for topological coincidences of co-diversification [22] due to the promiscuous host association in Aphidini [21], [25], [26].

The most striking result from this study was that extant heteroecious species could not use their secondary hosts before the Oligocene, because their secondary hosts emerged between the Oligocene and Miocene (Table 5). In other words, there were large temporal differences between the occurrences of primary and secondary hosts. As von Dohlen et al. [21] discussed, secondary hosts such as grasses and dicotyledonous herbs were not the major elements of temperate plant communities in the North Hemisphere, at least until the Miocene. The host association of *Melanaphis* can be viewed as crucial evidence since the earliest origins of C4 grasses, including *Miscanthus*, likely occurred about 32 MYA during the Oligocene [101]. Moreover, most heteroecious species in *Rhopalosiphum* and *Schizaphis* have adapted to many C4 grasses as a secondary host, such as *Echinochloa*, *Panicum*, *Pennisetum*, *Setaria*, *Sorghum*, and *Zea*, and they also utilize relatively young C3 grasses such as *Phragmites* and *Oryza* [14], [101]. The divergence times of heteroecious aphid genera were more congruent with the diversification of the primary hosts. It is tempting to conclude that the origin of species-level diversification coincided with the occurrences of the secondary hosts. However, more studies across diverse genera are needed to generalize the association of the species-level diversification in heteroecious aphids and their secondary hosts.

Our study also supports the multiple origins of host alternation [4], [16] within Aphidini. Von Dohlen [21] suggested that host alternation originated independently from Pterocommatinae, Macrosiphini, and Aphidini. However, our results differ from this basic premise in that Aphidini might have originated from monoecious ancestors. In the basal positions of Aphidina, three *Protaphis*-like species, *Aphis terricola*, *A. coprosmae*, and *A.crinosa*, likely diverged before the Oligocene and are monoecious with holocycly [14]. Instead, host alternation evolved further down the phylogeny independently in Rhopalosiphina and Aphidina. The likelihood of host-alternating origins in Aphidini (Figure 3; nodes A) is very low, whereas the group alternating between *Prunus* and Poaceae (node C) and the group alternating between *Ribes* and Onagraceae (node G) had a likelihood over 0.5 for host alternation, which diverged after the Middle Oligocene. In addition, *Cavariella* might have acquired host alternation earlier as seen in its time of diversification, which is consistent with that of Salicaceae and Araliaceae or closely-related Apiaceae occurring in the Middle to Late Eocene. Thus, there might be at least four independent origins of host alternation in Pterocommatinae+Aphidinae in our study.

The hypothesis of multiple origins of host alternation could conflict with the idea of the partitioned migration of females and males in Aphidinae, i.e., a single origin of separate migration of sexual winged males and females (i.e., gynoparae) bearing wingless egg-laying females (i.e., oviparae) (see von Dohlen et al. [21]). However, von Dohlen et al. [21] suggested that partitioning of winged male versus wingless oviparous female embryos into different viviparous females could be a plesiomorphic trait for Aphidinae. Although the gynopara is specialized to return to its primary host using its sensory capabilities [105], it is still uncertain whether this morph is the evolutionary result of host alternation. Except for two generations required to produce sexual females, mating between winged males and wingless oviparous females produced by winged viviparous migrants (i.e., sexuparae) occurs in other related monoecious taxa, including Calaphidinae, Chitophorinae, Drepanosiphinae, and Lachninae [2], [36]. In this light, the separate migration was likely acquired upon divergence from these monoecious subfamilies, as *Cavariella* also has gynoparae [21]. However, the other host-alternating aphid groups (Anoeciinae, Eriosomatinae, and Hormaphidinae), which are apparently phylogenetically distant from Aphidinae [36], still have sexuparae that produce both male and female sexuals in the primary host [3]. Therefore, one wonders why these aphids have not evolved specialized gynoparae like those in Aphidinae, even though most species in these groups alternate primary and secondary hosts in a one-year life cycle [4]. It might be concluded either that the separate migration is either plesiomorphic in Aphidinae as a whole or is unrelated to host alternation, in which case the life-history trait of host alternation most likely arose several times within Aphidinae.

Possible selection pressures for the evolution of host alternation in the Oligocene included climate change [106], [107] in conjunction with the nutritional superiority of herbaceous hosts [102], [108]. The origin of host alternation likely occurred during the rise of secondary herbaceous plants after the Oligocene climate change [101]. During the Oligocene, temperature and CO2 levels decreased, shifting the global climate toward more arid conditions [106]. Herbaceous plants such as the C4 grasses became dominant [101]. Aphids alternating their primary and secondary hosts might have obtained better nutritional sources [102], [109]. Alternatively, evolution of host alternation could be explained by fundatrix constraint, enemy escape, bet hedging, or induced responses hypotheses [110]. Unlike the climate change hypothesis, these hypotheses explain the maintenance of host alternation in aphids, rather than the origin or timing of the host alternation trait.

### Biogeography of the Aphidini

The biogeographic origin of the three main lineages of aphids (nodes A, B, and D) were equivocal, although a European origin received the highest probabilities in these three nodes (Figure 4). Within Rhopalosiphina, geographic inconsistencies made the species within each clade unclear. However, within Aphidina, four lineages (F, H, K, L) likely originated in Europe, whereas the *gossypii* group (node I) was most likely of an Asian origin. This suggests that Asian endemic species (e.g., *Aphis clerodendri*, *A. egomae*, and *A. sumire*) most likely originated from a common ancestor of node I (which includes the *gossypii* group), whereas European endemic species (including the *fabae*+*craccivora*+*spiraecola* groups) likely originated from a common ancestor of node K. In addition, *Bursaphis* originated in the Neartic (node G), and the four Southern Hemisphere species originated from Australasia after diverging from the European lineage. Interestingly, based on this result, the classical morphological groups of *Aphis* (i.e., subgenera and *Aphis* species-groups) were possibly separated by geographic isolation within Aphidina [14], [26], [27], [30], [84], [85], [111].

Although the nodes received low likelihood scores, the European and Australasian regions were more likely the biogeographic origins for Aphidini than were the Asian or Neartic regions. It seems that *Aphis terricola* diverged earliest among all aphidine aphids within Aphidini, and all five Southern Hemisphere species diverged relatively early within Aphidina. This inference hinges largely on the geographic origin of the basal taxa. No extant species diverged earlier than the subgenus *Protaphis* (*A.* (*P.*) *terricola*) within Aphidini, based on morphological and molecular systematics [30], [84], [85]. Furthermore, the two other *Protaphis*-like species, *A. coprosmae* and *A. crinosa*, subsequently diverged after the divergence of *A. terricola*, and the genus *Melanaphis* resembles many aphids in *Protaphis*, which is the basal group of Rhopalosiphina [26], [84], [85], [88]. It is rather interesting that the three species, *A. trerricola*, *A. coporosmae*, and *A. crinosa*, endemic to separate regions, appear in basal positions on the phylogeny in spite of the geographic gaps between them [14], [88]. To confirm the biogeographic origin of aphidine aphids, more research on phylogenies including more *Protaphis* and *Protaphis*-like species should be performed.

## Supporting Information

Figure S1 **Calibration points used as fixation or constraint of nodes for estimation of divergence times.**
(EPS)

Table S1 Aphid species used in this study with GenBank accession numbers, voucher numbers, and reference. Classification following Remaudière and Remaudière [24].
(DOC)

Table S2 Primers used for DNA amplification and sequencing.
(DOC)

Table S3 Life cycles, host types, and host plants of the Aphidini aphids.
(DOC)

Table S4 Biogeographic origin of the Aphidini aphids.
(DOC)

Table S5 Estimated divergence times of all nodes. The node numbers are those of Figures 1 and 2.
(DOC)
