# Supplementary material for: Macroevolutionary Patterns in the Aphidini Aphids (Hemiptera: Aphididae): Diversification, Host Association, and Biogeographic Origins
Source: PLoS One. 2011 Sep 15;6(9):e24749. doi: 10.1371/journal.pone.0024749 (PMC3174202; doi:10.1371/journal.pone.0024749)
Supplement: Table S1 — Aphid species used in this study with GenBank accession numbers, voucher numbers, and reference. Classification following Remaudière and Remaudière [24]. (DOC) [file pone.0024749.s002.doc]

**Table S1.** Aphid species used in this study with GenBank accession numbers, voucher numbers, and reference. Classification following Remaudière and Remaudière 1997 [24]

| Subfamily (Tribe) | Species | COI | tRNA/COII | CytB | 12S/16S | EF1a | Voucher no ^a^ or reference ^b^ |
| --- | --- | --- | --- | --- | --- | --- | --- |
| Aphidinae (Aphidini) | *Aphis* (*Aphis*) *acetosae* Linnaeus 1761 | - | AM085420 | AM085375 | - | - | A |
|  | *Aphis* (*Aphis*) *affinis* del Guercio 1911 | - | AM085412 | AM085367 | - | - | A |
|  | *Aphis* (*Aphis*) *arbuti* Ferrari 1872 | - | AM085428 | AM085383 | - | - | A |
|  | *Aphis* (*Aphis*) *argrimoniae* (Shinji 1941) | GQ904080 | GQ904149 | GU205342 | GU205375 | GU205373 | 080925HJ29 |
|  | *Aphis* (*Aphis*) *armata* Hausmann 1802 | - | AM085430 | AM085385 | - | - | A |
|  | *Aphis* (*Aphis*) *chloris* Koch 1854 | - | AM085411 | AM085366 | - | - | A |
|  | *Aphis* (*Aphis*) *clerodendri* Matsumura 1917 | GQ904081 | EU358824 | GU205343 | EU358864 | EU358904 | 031009SH32 |
|  | *Aphis* (*Aphis*) *coprosmae* Laing ex Tillyard 1926 | EU701300 | AY219750 | - | - | AY219729 | B, C |
|  | *Aphis* (*Aphis*) *coronillae* Ferrari 1872 | - | AM085419 | AM085374 | - | - | A |
|  | *Aphis* (*Aphis*) *cottieri* Carver 2000 | EU701304 | AY219751 | - | - | AY219730 | B, C |
|  | *Aphis* (*Aphis*) *craccae* Linnaeus 1758 | EU701307 | AM085421 | AM085376 | - | - | A, B |
|  | *Aphis* (*Aphis*) *craccivora* Koch 1854 | GQ904082 | EU358825 | GU205344 | EU358865 | EU358905 | 031026HJ1 |
|  | *Aphis* (*Aphis*) *crinosa* Paik 1969 | GQ904083 | EU358826 | GU205345 | EU358866 | EU358906 | 050425SH1 |
|  | *Aphis* (*Aphis*) *cytisorum* Hartig 1841 | - | AM085423 | AM085378 | - | - | A |
|  | *Aphis* (*Aphis*) *egomae* Shinji 1922 | GQ904084 | EU358827 | GU205346 | EU358867 | EU358907 | 050809HJ1 |
|  | *Aphis* (*Aphis*) *euphorbiae* Kaltenbach 1843 | - | AM085424 | AM085379 | - | - | A |
|  | *Aphis* (*Aphis*) *fabae* Scopoli 1763 | GQ904085 | EU358828 | GU205347 | EU358868 | EU358908 | 040527HJ16 |
|  | *Aphis* (*Aphis*) *frangulae* Kaltenbach 1845 | EU930142 | AM085407 | AM085362 | - | - | A, D |
|  | *Aphis* (*Aphis*) *fukii* Shinji 1922 | GQ904087 | EU358830 | GU205349 | EU358870 | EU358910 | 040527HJ10 |
|  | *Aphis* (*Aphis*) *glycines* Matsumura 1917 | GQ904089 | EU358831 | GU205350 | EU358871 | EU358911 | 040917HJ3 |
|  | *Aphis* (*Aphis*) *gossypii* Glover 1877 | GQ904096 | EU358832 | GU205351 | EU358872 | EU358912 | 030513HJ47 |
|  | *Aphis* (*Aphis*) *gossypii* type 1 [on *Rhamnus*] | GQ904131 | GQ904174 | GU457810 | GU457830 | GU457838 | 051025SH1 |
|  | *Aphis* (*Aphis*) *gossypii* type 2 [on *Rhamnus*] | GQ904132 | GQ904176 | GU457811 | GU457831 | GU457839 | 080925HJ16 |
|  | *Aphis* (*Aphis*) *healyi* Cottier 1953 | EU701423 | AY219752 | - | - | AY219731 | B, C |
|  | *Aphis* (*Aphis*) *hederae* Kaltenbach 1843 | GQ904101 | EU358833 | GU205352 | EU358873 | EU358913 | 060407SH24 |
|  | *Aphis* (*Aphis*) *helianthi* Monell in Riley & Monell 1879 | EU701434 | AY219744 | - | - | - | B, C |
|  | *Aphis* (*Aphis*) *hypericiphaga* Pashshenko 1933 | GQ904103 | EU358835 | GU205354 | EU358875 | EU358915 | 050616SH29 |
|  | *Aphis* (*Aphis*) *hypochoeridis* (Börner 1940) | - | AM085406 | AM085361 | - | - | A |
|  | *Aphis* (*Aphis*) *ichigo* Shinji 1922 | GQ904104 | EU358836 | GU205355 | EU358876 | EU358916 | 030625SH53 |
|  | *Aphis* (*Aphis*) *ichigocola* Shinji 1924 | GQ904105 | EU358837 | GU205356 | EU358877 | EU358917 | 030513SH10 |
|  | *Aphis* (*Aphis*) *idaei* van der Goot 1912 | EU701440 | AM085413 | AM085368 | - | - | A, B |
|  | *Aphis* (*Aphis*) *intybi* Koch 1855 | - | AM085418 | AM085373 | - | - | A |
|  | *Aphis* (*Aphis*) *jacobaeae* Schrank 1801 | - | AM085425 | AM085380 | - | - | A |
|  | *Aphis* (*Aphis*) *kurosawai* Takahashi 1921 | GQ904106 | EU358838 | GU205357 | EU358878 | EU358918 | 050603HJ16 |
|  | *Aphis* (*Aphis*) *neospiraeae* Takahashi 1966 | GQ904107 | EU358839 | GU205358 | EU358879 | EU358919 | 030523SH25 |
|  | *Aphis* (*Aphis*) *newtoni* Theobald 1927 | GQ904109 | EU358841 | GU205360 | EU358881 | EU358921 | 050603SH10 |
|  | *Aphis* (*Aphis*) *rumicis* Linnaeus 1758 | GQ904110 | EU358843 | GU205361 | EU358883 | EU358923 | 040513HJ10 |
|  | *Aphis* (*Aphis*) *salviae* Walker 1852 | - | AM085422 | AM085377 | - | - | A |
|  | *Aphis* (*Aphis*) *sanguisorbicola* Takahashi 1966 | GQ904111 | EU358844 | GU205362 | EU358884 | EU358924 | 040917HJ6 |
|  | *Aphis* (*Aphis*) *sedi* Kaltenbach 1843 | GQ904112 | GQ904161 | GU205363 | GU205376 | GU205374 | 030511HJ1 |
|  | *Aphis* (*Aphis*) *spiraecola* Patch 1914 | GQ904113 | EU358845 | GU205364 | EU358885 | EU358925 | 050603HJ6 |
|  | *Aphis* (*Aphis*) *sumire* Moritsu 1949 | GQ904114 | EU358846 | GU205365 | EU358886 | EU358926 | 050513HJ21 |
|  | *Aphis* (*Aphis*) *taraxacicola* (Börner 1940) | GQ904115 | EU358847 | GU205366 | EU358887 | EU358927 | 050513HJ11 |
|  | *Aphis* (*Aphis*) *teucrii* (Börner 1942) | - | AM085409 | AM085364 | - | - | A |
|  | *Aphis* (*Aphis*) *ulmariae* Schrank 1801 | - | AM085414 | AM085369 | - | - | A |
|  | *Aphis* (*Aphis*) *veratri* Walker 1852 | - | AM085427 | AM085382 | - | - | A |
|  | *Aphis* (*Aphis*) *viburni* Scopoli 1763 | - | AM085429 | AM085384 | - | - | A |
|  | *Aphis* (*Aphis*) sp.1 ex *Rhamnus* | GQ904135 | GQ904179 | GU457812 | GU457832 | GU457840 | 080925HJ41 |
|  | *Aphis* (*Aphis*) sp.2 ex *Rhamnus* | GQ904148 | GQ904192 | GU457813 | GU457833 | GU457841 | 080519HJ1 |
|  | *Aphis* (*Bursaphis*) *epilobii* Kaltenbach 1843 | - | AM085396 | AM085351 | - | - | A |
|  | *Aphis* (*Bursaphis*) *grossulariae* Kaltenbach 1843 | - | AM085400 | AM085355 | - | DQ418851 | A, E |
|  | *Aphis* (*Bursaphis*) *oenotherae* Oestlund 1887 | GQ904116 | EU358842 | GU205367 | EU358882 | EU358922 | 030625SH67 |
|  | *Aphis* (*Bursaphis*) *schneideri* (Börner 1940) | - | AM085399 | AM085354 | - | DQ418848 | A, E |
|  | *Aphis* (*Protaphis*) *terricola* Rondani 1848 | - | AM085395 | AM085350 | - | - | A |
|  | *Aphis* (*Toxopterina*) *vandergooti* (Börner 1939) | - | AM085394 | AM085349 | - | - | A |
|  | *Casimira* sp. | - | AY219742 | - | - | AY219722 | C |
|  | *Paradoxaphis aristoteliae* Sunde 1988 | EU701829 | AY219753 | - | - | AY219732 | B, C |
|  | *Paradoxaphis plagianthi* Eastop 2001 | EU701830 | AY219754 | - | - | AY219733 | B, C |
|  | *Toxoptera aurantii* (Boyer de Fonscolombe 1841) | GU457790 | EU358860 | GU457814 | EU358900 | EU358940 | 030513SH107 |
|  | *Euschizaphis* sp.1 | - | AY219741 | - | - | AY219721 | C |
|  | *Euschizaphis* sp.2 | - | AY219743 | - | - | AY219723 | C |
|  | *Hyalopterus pruni* (Geoffroy 1762) | GU457791 | EU358850 | GU457815 | EU358890 | EU358930 | 030424HJ2 |
|  | *Melanaphis japonica* (Takahashi 1919) | GU457792 | EU358852 | GU457816 | EU358892 | EU358932 | 030523SH29 |
|  | *Melanaphis luzulella* Hille Ris Lambers 1947 | - | AM085392 | AM085347 | - | - | A |
|  | *Rhopalosiphum insertum* Walker 1849 | EU701888 | AM085393 | AM085348 | - | - | A, B |
|  | *Rhopalosiphum maidis* (Fitch 1856) | GU457793 | EU358854 | GU457817 | EU358894 | EU358934 | 030625SH59 |
|  | *Rhopalosiphum nymphaeae* (Linnaeus 1761) | GU457794 | EU358855 | GU457818 | EU358895 | EU358935 | 050815HJ1 |
|  | *Rhopalosiphum padi* (Linnaeus 1758) | GU457795 | EU358856 | GU457819 | EU358896 | EU358936 | 030513SH2 |
|  | *Rhopalosiphum rufiabdominale* (Sasaki 1899) | GU457796 | EU358857 | GU457820 | EU358897 | EU358937 | 030531SH1 |
|  | *Schizaphis* (*Paraschizaphis*) *scirpi* (Passerini 1874) | GU457797 | EU358859 | GU457821 | EU358899 | EU358939 | 050804HJ1 |
|  | *Schizaphis* (*Schizaphis*) *graminum* (Rondani 1852) | GQ904117 | EU358858 | GU205372 | EU358898 | EU358938 | 050804HJ2 |
| Aphidinae ( Macrosiphini) | *Acyrthosiphon pisum* (Harris 1776) | EU071328 | EU071348 | EU071338 | EU071318 | EU071358 | 030513SH39 |
|  | *Brevicoryne brassicae* (Linnaeus 1758) | GU457798 | EU358848 | GU457822 | EU358888 | EU358928 | 040513HJ27 |
|  | *Cavariella salicicola* (Matsumura 1917) | GU457799 | GU457806 | GU457823 | GU457834 | GU457842 | 070518HJ34 |
|  | *Cryptosiphum artemisiae* Buckton 1879 | GU457800 | EU358849 | GU457824 | EU358889 | EU358929 | 040527HJ26 |
|  | *Lipaphis pseudobrassicae* (Davis 1914) | GU457801 | EU358851 | GU457825 | EU358891 | EU358931 | 050422SH7 |
|  | *Megoura crassicauda* Mordvilko 1919 | EU071319 | EU071339 | EU071329 | EU071309 | EU071349 | 030513SH04 |
|  | *Myzus* *persicae* (Sulzer 1776) | GU457802 | EU358853 | GU457826 | EU358893 | EU358933 | 050502HJ4 |
| Pterocommatinae | *Pterocomma pilosum* Buckton 1879 | GU457803 | GU457807 | GU457827 | GU457835 | GU457843 | 030523SH15 |
|  | *Pterocomma populifoliae* Palmer 1952 | - | DQ005183 | - | AF275251 | DQ005139 | C, F |
| Hormaphidinae ^c^ | *Hamamelistes spinosus* Shimer 1867 | EF073057 | AF454619 | - | AF275226 | AF545607 | C, G |
|  | *Nipponaphis coreanus* (Paik 1965) | GU457804 | GU457808 | GU457828 | GU457836 | GU457844 | 030513SH104 |
| Lachninae ^c^ | *Cinara longipennis* (Matsumura 1917) | GU457805 | GU457809 | GU457829 | GU457837 | GU457845 | 050423SH1 |
| Eriosomatinae ^c^ | *Melaphis rhois* (Fitch 1866) | EU701748 | FJ215686 | AY601894 | AF275223 | FJ215686 | B, C, F, H |
|  | *Schlechtendalia chinensis* (Bell 1851) | - | AF454628 | AY601893 | AF275222 | EU363670 | F, H, I |
| Adelgidae ^c, d^ | *Adelges cooleyi* (Gillette 1907) | EF073065 | EF073127 | EF073185 | AF275216 | EF073224 | F, G |
| Phylloxeridae ^c, d^ | *Phylloxera* sp. | EF073060 | EF073122 | EF073184 | - | EF073222 | G |

a: samples collected from the central and southern regions of the Korean Peninsula during 2003-2007. All voucher specimens (colony individuals) deposited in Seoul National University, Rep. of Korea.

b: reference sequences form previous studies: (A) Coeur d'acier et al. 2007 [30]; (B) Foottit et al. 2008 [23]; (C) von Dohlen and Teulon 2003 [25]; (D) Carletto et al. 2009 [32]; (E) Turcinaviciene et al. 2006 [29]; (F) von Dohlen and Moran 2000 [16]; (G) Havill et al. 2007 [22]; (H) Thao et al. 2004 [31]; (I) Yang et al. 2010 [33]

c: outgroup

d: family
