# Supplementary material for: Macroevolutionary Patterns in the Aphidini Aphids (Hemiptera: Aphididae): Diversification, Host Association, and Biogeographic Origins
Source: PLoS One. 2011 Sep 15;6(9):e24749. doi: 10.1371/journal.pone.0024749 (PMC3174202; doi:10.1371/journal.pone.0024749)
Supplement: Table S2 — Primers used for DNA amplification and sequencing. (DOC) [file pone.0024749.s003.doc]

**Table S2.** Primers used for DNA amplification and sequencing

| Gene Location | Primer name | Sequence | Reference |
| --- | --- | --- | --- |
| COI | LCO1490 | 5'-GGTCAACAAATCATAAAGATATTGG-3' | Folmer et al. 1994 [39] |
|  | HCO2198 | 5'-TAAACTTCAGGGTGACCAAAAAATCA-3' | `` |
| tRNA/COII | 2993+ | 5'-CATTCATATTCAGAATTACC-3' | Stern 1994 [40] |
|  | A3772 | 5'-GAGACCATTACTTGCTTTCAGTCATCT-3' | Normark 1996 [41] |
| CytB | F18 | 5'-GATGATGAAATTTTGGAT-3' | Harry et al. 1998 [42] |
|  | R18 | 5'-CTAATGCAATAACTCCTC-3' | `` |
|  | CB2 | 5'-ATTACACCTCCTAATTTATTAGGAAT-3' | Jermiin and Crozier 1994 [43] |
| 12S/16S | 12Sai | 5'-AAACTAGGATTAGATACCCTATTAT-3' | Simon et al. 1994 [44] |
|  | 12Sfr* | 5'-AAATATGTACATATTGCCCG-3' | Simon et al. 1991 [45] |
|  | 1470a* | 5'-TAGTACCTTTTGTATCAGGG-3' | von Dohlen and Moran 2000 [16] |
|  | 1473* | 5'-AGTTTTATAGGGTCTTATCGTC-3' | `` |
|  | 1472 | 5'-AGATAGAAACCAACCTGG-3' | `` |
| EF1α | EF2 | 5'-ATGTGAGCAGTGTGGCAATCCAA-3' | Palumbi 1996 [46] |
|  | EF3 | 5'-GAACGTGAACGTGGTATCAC-3' | von Dohlen et al. 2002 [47] |
|  | EF6 | 5'-TGACCAGGGTGGTTCAATAC-3' | `` |

* Internal primer
