# Supplementary material for: Macroevolutionary Patterns in the Aphidini Aphids (Hemiptera: Aphididae): Diversification, Host Association, and Biogeographic Origins
Source: PLoS One. 2011 Sep 15;6(9):e24749. doi: 10.1371/journal.pone.0024749 (PMC3174202; doi:10.1371/journal.pone.0024749)
Supplement: Table S3 — Life cycles, host types, and host plants of the Aphidini aphids. (DOC) [file pone.0024749.s004.doc]

**Table S3.** Life cycle, host type, and host plant of the Aphidini aphids

| Subtribe | Species | Life cycle & host type | Host plant ^a^ | Reference ^b^ |
| --- | --- | --- | --- | --- |
| Aphidina | *Aphis* (*Aphis*) *acetosae* Linnaeus 1761 | Monoecy on grasses | *Rumex* spp*.* | A, B, E |
|  | *Aphis* (*Aphis*) *affinis* del Guercio 1911 | Monoecy on grasses | *Mentha* spp*.* | A, B, E |
|  | *Aphis* (*Aphis*) *arbuti* Ferrari 1872 | Monoecy on trees | Ericaceae | A, B |
|  | *Aphis* (*Aphis*) *argrimoniae* (Shinji 1941) | Monoecy on grasses | *Agrimonia pilosa* | A, C |
|  | *Aphis* (*Aphis*) *armata* Hausmann 1802 | Monoecy on grasses | *Digitalis* spp*.* | A, B |
|  | *Aphis* (*Aphis*) *chloris* Koch 1854 | Monoecy on grasses | *Hypericum* spp*.* | A, B |
|  | *Aphis* (*Aphis*) *clerodendri* Matsumura 1917 | Monoecy on trees | *Clerodendron* spp*.* | A, C |
|  | *Aphis* (*Aphis*) *coprosmae* Laing ex Tillyard 1926 | Monoecy on trees | *Coporosma* spp*.* | A, D, F |
|  | *Aphis* (*Aphis*) *coronillae* Ferrari 1872 | Monoecy on grasses | Fabaceae (3 genera) | A, B, E |
|  | *Aphis* (*Aphis*) *cottieri* Carver 2000 | Monoecy on trees | *Muehlenbeckia* spp. | A, D |
|  | *Aphis* (*Aphis*) *craccae* Linnaeus 1758 | Monoecy on grasses | *Vicia cracca* | A, B, E |
|  | *Aphis* (*Aphis*) *craccivora* Koch 1854 | Monoecy on trees | Polyphagous within Fabaceae | A, B, C, E |
|  | *Aphis* (*Aphis*) *crinosa* Paik 1969 | Monoecy on trees | *Ligstrum* (*foliosum, obtusifolium*) | A, C |
|  | *Aphis* (*Aphis*) *cytisorum* Hartig 1841 | Monoecy on trees | Fabaceae (3 genera) | A, B, E |
|  | *Aphis* (*Aphis*) *egomae* Shinji 1922 | Monoecy on grasses | *Perilla frutescens* | A, C |
|  | *Aphis* (*Aphis*) *euphorbiae* Kaltenbach 1843 | Monoecy on grasses | *Euphorbia* spp*.* | A, B |
|  | *Aphis* (*Aphis*) *fabae* Scopoli 1763 | Heteroecy | [P.] *Euonymus, Viburnum*, [S.] Polyphagous to more than 5 plant families | A, B, C, E |
|  | *Aphis* (*Aphis*) *frangulae* Kaltenbach 1845 | Heteroecy | [P.] *Rhamnus, Frangula*, [S.] *Capsella, Lysimachia, Epilobium* | A, B, E |
|  | *Aphis* (*Aphis*) *fukii* Shinji 1922 | Monoecy on grasses | *Petasites japonicus* | A, C |
|  | *Aphis* (*Aphis*) *glycines* Matsumura 1917 | Heteroecy | [P.] *Rhamnus* (*davurica, japonica*), [S.] *Glycine max* | A, C |
|  | *Aphis* (*Aphis*) *gossypii* Glover 1877 | Heteroecy | [P.] *Rhamnus*, *Hibiscus*, [S.] Polyphagous to more than 20 plant families | A, B, C, E |
|  | *Aphis* (*Aphis*) *gossypii* type 1 [on *Rhamnus*] | Heteroecy | [P.] *Rhamnus*, *Hibiscus*, [S.] Polyphagous to more than 20 plant families | A, B, C, E |
|  | *Aphis* (*Aphis*) *gossypii* type 2 [on *Rhamnus*] | Heteroecy | [P.] *Rhamnus*, *Hibiscus*, [S.] Polyphagous to more than 20 plant families | A, B, C, E |
|  | *Aphis* (*Aphis*) *healyi* Cottier 1953 | Monoecy on trees | *Carmichaelia* spp*.* | A, D, F |
|  | *Aphis* (*Aphis*) *hederae* Kaltenbach 1843 | Monoecy on trees | *Hedera* spp*.* | A, C, E |
|  | *Aphis* (*Aphis*) *helianthi* Monell in Riley & Monell 1879 | Monoecy on grasses | [P.] *Cornus*, [S.] Apiaceae, Asteraceae | A, D, F |
|  | *Aphis* (*Aphis*) *hypericiphaga* Pashshenko 1933 | Monoecy on grasses | *Hypericum* spp*.* | A, C |
|  | *Aphis* (*Aphis*) *hypochoeridis* (Börner 1940) | Monoecy on grasses | *Hypochaeris* spp*.* | A, B, E |
|  | *Aphis* (*Aphis*) *ichigo* Shinji 1922 | Monoecy on trees | *Rubus* spp. (5 species) | A, C |
|  | *Aphis* (*Aphis*) *ichigocola* Shinji 1924 | Monoecy on trees | *Rubus* spp. (3 species) | A, C |
|  | *Aphis* (*Aphis*) *idaei* van der Goot 1912 | Monoecy on trees | *Rubus* spp. (4 species) | A, B, E |
|  | *Aphis* (*Aphis*) *intybi* Koch 1855 | Monoecy on grasses | *Cichorium intybus* | A, B, E |
|  | *Aphis* (*Aphis*) *jacobaeae* Schrank 1801 | Monoecy on grasses | *Senecio jacobea* | A, B, E |
|  | *Aphis* (*Aphis*) *kurosawai* Takahashi 1921 | Monoecy on grasses | *Artemisia* spp. (11 species) | A, C |
|  | *Aphis* (*Aphis*) *neospiraeae* Takahashi 1966 | Monoecy on trees | *Spiraea* spp*.* | A, C |
|  | *Aphis* (*Aphis*) *newtoni* Theobald 1927 | Monoecy on grasses | *Iris* spp*.* | A, C, E |
|  | *Aphis* (*Aphis*) *rumicis* Linnaeus 1758 | Monoecy on grasses | *Rumex* spp*.* | A, C, E |
|  | *Aphis* (*Aphis*) *salviae* Walker 1852 | Monoecy on grasses | *Salvia* spp*.* | A, B |
|  | *Aphis* (*Aphis*) *sanguisorbicola* Takahashi 1966 | Monoecy on grasses | *Sanguisorba* spp. (4 species) | A, C |
|  | *Aphis* (*Aphis*) *sedi* Kaltenbach 1843 | Monoecy on grasses | Crassulaceae (*Sedum*, *Sempervivum*) | A, C, E |
|  | *Aphis* (*Aphis*) *spiraecola* Patch 1914 | Heteroecy | [P.] *Spirea*, *Citrus*, [S.] Polyphagous to more than 20 plant families | A, B, C, E |
|  | *Aphis* (*Aphis*) *sumire* Moritsu 1949 | Monoecy on grasses | *Viola* spp*.* | A, C |
|  | *Aphis* (*Aphis*) *taraxacicola* (Börner 1940) | Monoecy on grasses | *Taraxacum* spp*.* | A, C, E |
|  | *Aphis* (*Aphis*) *teucrii* (Börner 1942) | Monoecy on trees | *Teucrium* spp. | A, B |
|  | *Aphis* (*Aphis*) *ulmariae* Schrank 1801 | Monoecy on grasses | *Filipendula* (*palmata, ulamaria*) | A, B, C, E |
|  | *Aphis* (*Aphis*) *veratri* Walker 1852 | Monoecy on grasses | *Veratrum* spp*.* | A, B |
|  | *Aphis* (*Aphis*) *viburni* Scopoli 1763 | Monoecy on grasses | *Viburnum* spp*.* | A, B, E |
|  | *Aphis* (*Aphis*) sp.1 ex *Rhamnus* | Heteroecy | [P.] *Rhamnus davurica*, [S.] unknown | A, C, G |
|  | *Aphis* (*Aphis*) sp.2 ex *Rhamnus* | Heteroecy | [P.] *Rhamnus davurica*, [S.] unknown | A, C, G |
|  | *Aphis* (*Bursaphis*) *epilobii* Kaltenbach 1843 | Monoecy on grasses | *Epilobium* spp*.* | A, B, E |
|  | *Aphis* (*Bursaphis*) *grossulariae* Kaltenbach 1843 | Heteroecy | [P.] *Ribes grossularia*, [S.] Onagraceae (*Epilobium*, *Clarkia*) | A, E |
|  | *Aphis* (*Bursaphis*) *oenotherae* Oestlund 1887 | Heteroecy | [P.] *Ribes* spp. (3 species), [S.] Onagraceae (*Epilobium*, *Oenothera*) | A, C, E |
|  | *Aphis* (*Bursaphis*) *schneideri* (Börner 1940) | Monoecy on trees | *Ribes nigrum* | A, E |
|  | *Aphis* (*Protaphis*) *terricola* Rondani 1848 | Monoecy on grasses | *Centaurea* spp*.* | A, B |
|  | *Aphis* (*Toxopterina*) *vandergooti* (Börner 1939) | Monoecy on trees | Anthemidae | A, B |
|  | *Casimira* sp. | Monoecy on grasses | *Ozothamnus leptophyllus* | A, F |
|  | *Paradoxaphis aristoteliae* Sunde 1988 | Monoecy on trees | *Aristotelia serrata* | A, D, F |
|  | *Paradoxaphis plagianthi* Eastop 2001 | Monoecy on trees | *Plagianthus regius* | A, D, F |
|  | *Toxoptera aurantii* (Boyer de Fonscolombe 1841) | Anholocycly* | Anacardiaceae, Anonaceae, Araliaceae, Euphorbiaceae, Lauraceae, Moraceae, Rubiaceae, Rutaceae, Sterculiaceae, Theaceae | A, C |
|  | *Euschizaphis* sp.1 | Monoecy on trees | *Dracophyllum* spp*.* | A, F |
|  | *Euschizaphis* sp.2 | Monoecy on grasses | *Aciphylla aurea* | A, F |
| Rhopalosiphina | *Hyalopterus pruni* (Geoffroy 1762) | Heteroecy | [P.] *Prunus* spp. (5 species), [S.] *Phragmites* (*communis, longivalvis*) | A, C, E |
|  | *Melanaphis japonica* (Takahashi 1919) | Monoecy on grasses | *Miscanthus sinensis* | A, C |
|  | *Melanaphis luzulella* Hille Ris Lambers 1947 | Monoecy on grasses | *Luzula* spp. | A, B, E |
|  | *Rhopalosiphum insertum* Walker 1849 | Heteroecy | [P.] *Chaenomeles, Cotoneaster, Cydoni*, [S.] Poaceae (9 genera) | A, B, E |
|  | *Rhopalosiphum maidis* (Fitch 1856) | Heteroecy | [P.] *Prunus* spp. (3 species), [S.] Poaceae (10 genera) | A, C, E |
|  | *Rhopalosiphum nymphaeae* (Linnaeus 1761) | Heteroecy | [P.] *Prunus* spp. (15 species), [S.] *Alisma, Nuphar, Nelumbo, Nymphaea* | A, C, E |
|  | *Rhopalosiphum padi* (Linnaeus 1758) | Heteroecy | [P.] *Prunus* spp. (15 species), [S.] Poaceae, Acoraceae, Cyperaceae | A, C, E |
|  | *Rhopalosiphum rufiabdominale* (Sasaki 1899) | Heteroecy | [P.] *Prunus* spp. (8 species), [S.] Poaceae (3 genera) | A, C, E |
|  | *Schizaphis* (*Paraschizaphis*) *scirpi* (Passerini 1874) | Monoecy on grasses | *Typha, Sparganium,* Cyperaceae | A, C, E |
|  | *Schizaphis* (*Schizaphis*) *graminum* (Rondani 1852) | Monoecy on grasses | Poaceae, Cyperaceae | A, C |

a: [P.] = primary host; [S.] = secondary host

b: reference for life cycle and host plant: (A) Coeur d'acier et al. 2007 [30]; (B) Foottit et al. 2008 [23]; (C) von Dohlen and Teulon 2003 [25]; (D) Carletto et al. 2009 [32]; (E) Turcinaviciene et al. 2006 [29]; (F) von Dohlen and Moran 2000 [16]; (G) Kim et al. 2010 [27]

* considered as monoecious holocyclic in reconstructing the ancestral state of host alternation
