# Supplementary material for: Macroevolutionary Patterns in the Aphidini Aphids (Hemiptera: Aphididae): Diversification, Host Association, and Biogeographic Origins
Source: PLoS One. 2011 Sep 15;6(9):e24749. doi: 10.1371/journal.pone.0024749 (PMC3174202; doi:10.1371/journal.pone.0024749)
Supplement: Table S4 — Biogeographic origin of the Aphidini aphids. (DOC) [file pone.0024749.s005.doc]

**Table S4.** Biogeogrphic origin of the Aphidini aphids

| Subtribe | Species | Biogeographic origin ^a, b^ | Reference ^c^ |
| --- | --- | --- | --- |
| Aphidina | *Aphis* (*Aphis*) *acetosae* Linnaeus 1761 | AB | A, B, E |
|  | *Aphis* (*Aphis*) *affinis* del Guercio 1911 | A | A, B, E |
|  | *Aphis* (*Aphis*) *arbuti* Ferrari 1872 | A | A, B |
|  | *Aphis* (*Aphis*) *argrimoniae* (Shinji 1941) | B | A, C |
|  | *Aphis* (*Aphis*) *armata* Hausmann 1802 | A | A, B |
|  | *Aphis* (*Aphis*) *chloris* Koch 1854 | A | A, B |
|  | *Aphis* (*Aphis*) *clerodendri* Matsumura 1917 | B | A, C |
|  | *Aphis* (*Aphis*) *coprosmae* Laing ex Tillyard 1926 | C | A, D, F |
|  | *Aphis* (*Aphis*) *coronillae* Ferrari 1872 | A | A, B, E |
|  | *Aphis* (*Aphis*) *cottieri* Carver 2000 | C | A, D |
|  | *Aphis* (*Aphis*) *craccae* Linnaeus 1758 | AB | A, B, E |
|  | *Aphis* (*Aphis*) *craccivora* Koch 1854 | ABCD | A, B, C, E |
|  | *Aphis* (*Aphis*) *crinosa* Paik 1969 | B | A, C |
|  | *Aphis* (*Aphis*) *cytisorum* Hartig 1841 | AD | A, B, E |
|  | *Aphis* (*Aphis*) *egomae* Shinji 1922 | B | A, C |
|  | *Aphis* (*Aphis*) *euphorbiae* Kaltenbach 1843 | A | A, B |
|  | *Aphis* (*Aphis*) *fabae* Scopoli 1763 | AD | A, B, C, E |
|  | *Aphis* (*Aphis*) *frangulae* Kaltenbach 1845 | A | A, B, E |
|  | *Aphis* (*Aphis*) *fukii* Shinji 1922 | B | A, C |
|  | *Aphis* (*Aphis*) *glycines* Matsumura 1917 | B | A, C |
|  | *Aphis* (*Aphis*) *gossypii* Glover 1877 | ABCD | A, B, C, E |
|  | *Aphis* (*Aphis*) *gossypii* type 1 [on *Rhamnus*] | B | A, B, C, E |
|  | *Aphis* (*Aphis*) *gossypii* type 2 [on *Rhamnus*] | B | A, B, C, E |
|  | *Aphis* (*Aphis*) *healyi* Cottier 1953 | C | A, D, F |
|  | *Aphis* (*Aphis*) *hederae* Kaltenbach 1843 | ABCD | A, C, E |
|  | *Aphis* (*Aphis*) *helianthi* Monell in Riley & Monell 1879 | A | A, D, F |
|  | *Aphis* (*Aphis*) *hypericiphaga* Pashshenko 1933 | B | A, C |
|  | *Aphis* (*Aphis*) *hypochoeridis* (Börner 1940) | A | A, B, E |
|  | *Aphis* (*Aphis*) *ichigo* Shinji 1922 | B | A, C |
|  | *Aphis* (*Aphis*) *ichigocola* Shinji 1924 | B | A, C |
|  | *Aphis* (*Aphis*) *idaei* van der Goot 1912 | A | A, B, E |
|  | *Aphis* (*Aphis*) *intybi* Koch 1855 | A | A, B, E |
|  | *Aphis* (*Aphis*) *jacobaeae* Schrank 1801 | A | A, B, E |
|  | *Aphis* (*Aphis*) *kurosawai* Takahashi 1921 | B | A, C |
|  | *Aphis* (*Aphis*) *neospiraeae* Takahashi 1966 | B | A, C |
|  | *Aphis* (*Aphis*) *newtoni* Theobald 1927 | AB | A, C, E |
|  | *Aphis* (*Aphis*) *rumicis* Linnaeus 1758 | ABD | A, C, E |
|  | *Aphis* (*Aphis*) *salviae* Walker 1852 | A | A, B |
|  | *Aphis* (*Aphis*) *sanguisorbicola* Takahashi 1966 | B | A, C |
|  | *Aphis* (*Aphis*) *sedi* Kaltenbach 1843 | ABCD | A, C, E |
|  | *Aphis* (*Aphis*) *spiraecola* Patch 1914 | ABCD | A, B, C, E |
|  | *Aphis* (*Aphis*) *sumire* Moritsu 1949 | B | A, C |
|  | *Aphis* (*Aphis*) *taraxacicola* (Börner 1940) | AB | A, C, E |
|  | *Aphis* (*Aphis*) *teucrii* (Börner 1942) | ABD | A, B |
|  | *Aphis* (*Aphis*) *ulmariae* Schrank 1801 | A | A, B, C, E |
|  | *Aphis* (*Aphis*) *veratri* Walker 1852 | A | A, B |
|  | *Aphis* (*Aphis*) *viburni* Scopoli 1763 | A | A, B, E |
|  | *Aphis* (*Aphis*) sp.1 ex *Rhamnus* | B | A, C, G |
|  | *Aphis* (*Aphis*) sp.2 ex *Rhamnus* | B | A, C, G |
|  | *Aphis* (*Bursaphis*) *epilobii* Kaltenbach 1843 | A | A, B, E |
|  | *Aphis* (*Bursaphis*) *grossulariae* Kaltenbach 1843 | A | A, E |
|  | *Aphis* (*Bursaphis*) *oenotherae* Oestlund 1887 | D | A, C, E |
|  | *Aphis* (*Bursaphis*) *schneideri* (Börner 1940) | A | A, E |
|  | *Aphis* (*Protaphis*) *terricola* Rondani 1848 | A | A, B |
|  | *Aphis* (*Toxopterina*) *vandergooti* (Börner 1939) | A | A, B |
|  | *Casimira* sp. | C | A, F |
|  | *Paradoxaphis aristoteliae* Sunde 1988 | C | A, D, F |
|  | *Paradoxaphis plagianthi* Eastop 2001 | C | A, D, F |
|  | *Toxoptera aurantii* (Boyer de Fonscolombe 1841) | ABCD | A, C |
|  | *Euschizaphis* sp.1 | C | A, F |
|  | *Euschizaphis* sp.2 | C | A, F |
| Rhopalosiphina | *Hyalopterus pruni* (Geoffroy 1762) | ABCD | A, C, E |
|  | *Melanaphis japonica* (Takahashi 1919) | B | A, C |
|  | *Melanaphis luzulella* Hille Ris Lambers 1947 | A | A, B, E |
|  | *Rhopalosiphum insertum* Walker 1849 | D | A, B, E |
|  | *Rhopalosiphum maidis* (Fitch 1856) | ABD | A, C, E |
|  | *Rhopalosiphum nymphaeae* (Linnaeus 1761) | ABCD | A, C, E |
|  | *Rhopalosiphum padi* (Linnaeus 1758) | AB | A, C, E |
|  | *Rhopalosiphum rufiabdominale* (Sasaki 1899) | ABCD | A, C, E |
|  | *Schizaphis* (*Paraschizaphis*) *scirpi* (Passerini 1874) | AB | A, C, E |
|  | *Schizaphis* (*Schizaphis*) *graminum* (Rondani 1852) | AB | A, C |

a: code of biogeographic origin used in BayesMultistate: (A) European (with some regions in the Western Palearcic), (B) Asian (with some regions in the Eastern Palearctic), (C) Australasian, (D) Neartic, (AB) Palearctic, (ABD) Palearctic + Nearctic, (ABCD) Cosmopolitan

b: multistate used when biogeographic origin of the species is equivocal among two or more regions

c: reference for life cycle and host plant: (A) Coeur d'acier et al. 2007 [30]; (B) Foottit et al. 2008 [23]; (C) von Dohlen and Teulon 2003 [25]; (D) Carletto et al. 2009 [32]; (E) Turcinaviciene et al. 2006 [29]; (F) von Dohlen and Moran 2000 [16]; (G) Kim et al. 2010 [27]
