# Supplementary material for: Macroevolutionary Patterns in the Aphidini Aphids (Hemiptera: Aphididae): Diversification, Host Association, and Biogeographic Origins
Source: PLoS One. 2011 Sep 15;6(9):e24749. doi: 10.1371/journal.pone.0024749 (PMC3174202; doi:10.1371/journal.pone.0024749)
Supplement: Table S5 — Estimated divergence times of all nodes. The node numbers are those of Figures 1 and 2. (DOC) [file pone.0024749.s006.doc]

**Table S5.** Estimated divergence times of all nodes. The node numbers are indicated in Figures 1 and 2 in main text

|  | BEAST | |  | MULTIDIVTIME | |
| --- | --- | --- | --- | --- | --- |
| node no. | time | 95% HDP range |  | time | 95% HDP range |
| II | 97.0 | (91.5-100.0) |  | 94.2 | (85.1-99.7) |
| III | 94.2 | (87.9-99.3) |  | 92.4 | (83.5-98.7) |
| IV | 65.7 | (48.5-82.5) |  | 70.8 | (57.5-84.0) |
| V | 83.7 | (73.9-93.4) |  | 87.7 | (77.3-96.4) |
| VI | 52.4 | (48.0-59.6) |  | 51.5 | (48.1-59.4) |
| 1 | 68.5 | (58.8-78.5) |  | 67.2 | (58.7-77.4) |
| 2 | 62.0 | (52.9-71.0) |  | 62.2 | (54.5-71.9) |
| 3 | 52.9 | (45.0-61.4) |  | 55.0 | (50.2-63.9) |
| 4 | 48.6 | (38.4-58.9) |  | 48.9 | (39.9-59.3) |
| 5 | 42.9 | (29.6-55.9) |  | 42.2 | (31.9-53.8) |
| 6 | 13.9 | (8.0-21.0) |  | 11.9 | (5.9-19.7) |
| 7 | 33.9 | (24.0-44.3) |  | 34.5 | (25.7-44.7) |
| 8 | 27.0 | (17.4-36.7) |  | 29.0 | (20.2-39.2) |
| 9 | 42.1 | (31.7-52.6) |  | 42.5 | (33.2-52.8) |
| 10 | 34.3 | (23.4-45.7) |  | 36.2 | (26.3-47.1) |
| 11 | 44.9 | (36.9-53.2) |  | 50.6 | (43.8-59.7) |
| 12 | 38.4 | (31.2-46.4) |  | 42.0 | (34.5-51.2) |
| 13 | 34.5 | (27.4-41.7) |  | 40.0 | (32.6-48.9) |
| 14 | 28.0 | (22.2-34.6) |  | 33.0 | (25.7-41.8) |
| 15 | 25.1 | (19.1-31.5) |  | 28.0 | (20.9-36.4) |
| 16 | 15.0 | (9.8-20.6) |  | 14.4 | (9.4-20.7) |
| 17 | 11.3 | (6.6-16.4) |  | 11.3 | (6.2-17.4) |
| 18 | 24.2 | (18.5-30.4) |  | 29.7 | (22.3-38.3) |
| 19 | 18.8 | (12.7-25.2) |  | 19.7 | (8.9-30.7) |
| 20 | 18.6 | (12.7-24.8) |  | 23.8 | (14.9-33.5) |
| 21 | 16.3 | (10.4-22.4) |  | 16.8 | (6.3-27.7) |
| 22 | 47.2 | (38.6-55.6) |  | 50.4 | (42.6-60.0) |
| 23 | 43.4 | (36.0-51.1) |  | 45.6 | (38.4-54.6) |
| 24 | 39.9 | (32.9-47.2) |  | 43.1 | (36.1-52.0) |
| 25 | 37.8 | (31.4-44.6) |  | 39.1 | (32.0-47.8) |
| 26 | 35.9 | (29.6-42.5) |  | 35.7 | (28.5-44.4) |
| 27 | 23.9 | (16.9-30.8) |  | 24.8 | (16.6-34.5) |
| 28 | 17.5 | (11.6-23.7) |  | 16.8 | (10.1-25.1) |
| 29 | 14.5 | (9.2-20.5) |  | 13.5 | (6.9-21.4) |
| 30 | 32.7 | (27.1-38.7) |  | 30.4 | (23.8-38.7) |
| 31 | 15.0 | (8.2-22.8) |  | 13.6 | (6.4-22.4) |
| 32 | 3.9 | (1.5-6.7) |  | 4.2 | (1.0-8.8) |
| 33 | 0.7 | (0.1-1.7) |  | 1.0 | (0.0-2.9) |
| 34 | 31.8 | (26.3-37.6) |  | 28.0 | (21.7-35.9) |
| 35 | 25.3 | (20.2-30.6) |  | 20.4 | (14.7-27.6) |
| 36 | 20.1 | (15.4-24.8) |  | 16.6 | (11.6-23.0) |
| 37 | 17.5 | (13.5-21.8) |  | 15.0 | (10.5-20.9) |
| 38 | 15.8 | (11.9-19.6) |  | 13.6 | (9.3-19.2) |
| 39 | 13.9 | (10.5-17.4) |  | 11.7 | (7.8-16.8) |
| 40 | 10.8 | (8.1-13.6) |  | 10.8 | (7.0-15.7) |
| 41 | 8.0 | (6.0-10.1) |  | 6.4 | (3.9-9.8) |
| 42 | 4.6 | (2.5-6.8) |  | 3.8 | (1.9-6.5) |
| 43 | 7.0 | (5.2-8.9) |  | 4.9 | (2.9-7.8) |
| 44 | 6.3 | (4.3-8.3) |  | 3.9 | (1.9-6.5) |
| 45 | 3.2 | (1.6-5.1) |  | 2.0 | (0.5-4.2) |
| 46 | 1.1 | (0.2-2.2) |  | 1.0 | (0.1-2.8) |
| 47 | 5.6 | (4.0-7.3) |  | 4.5 | (2.5-7.1) |
| 48 | 4.6 | (3.1-6.1) |  | 2.9 | (1.5-4.9) |
| 49 | 3.8 | (2.4-5.4) |  | 2.4 | (1.0-4.3) |
| 50 | 3.0 | (1.8-4.2) |  | 1.5 | (0.6-2.9) |
| 51 | 0.5 | (0.1-1.0) |  | 0.6 | (0.1-1.5) |
| 52 | 2.3 | (1.5-3.4) |  | 1.2 | (0.4-2.4) |
| 53 | 2.1 | (1.2-3.3) |  | 0.7 | (0.1-1.8) |
| 54 | 1.6 | (0.8-2.5) |  | 0.9 | (0.2-2.0) |
| 55 | 0.5 | (0.7-2.3) |  | 0.6 | (0.0-1.6) |
| 56 | 1.5 | (0.0-1.1) |  | 0.4 | (0.0-1.3) |
| 57 | 27.5 | (22.4-32.9) |  | 25.2 | (18.9-32.8) |
| 58 | 23.7 | (17.6-29.7) |  | 21.3 | (13.8-29.5) |
| 59 | 19.8 | (13.3-26.8) |  | 17.9 | (9.6-26.4) |
| 60 | 16.7 | (10.5-22.7) |  | 13.5 | (4.5-22.7) |
| 61 | 25.7 | (20.8-30.8) |  | 23.1 | (17.3-30.3) |
| 62 | 17.4 | (11.4-23.3) |  | 17.1 | (11.4-23.9) |
| 63 | 22.4 | (17.6-27.2) |  | 19.8 | (14.3-26.7) |
| 64 | 12.6 | (8.7-16.9) |  | 11.7 | (6.2-19.0) |
| 65 | 11.0 | (7.6-14.8) |  | 8.2 | (4.3-13.7) |
| 66 | 8.8 | (5.2-12.6) |  | 5.6 | (1.4-10.7) |
| 67 | 8.4 | (5.2-11.9) |  | 5.7 | (2.7-9.9) |
| 68 | 4.6 | (2.1-7.2) |  | 3.8 | (1.2-7.4) |
| 69 | 0.8 | (0.1-1.7) |  | 1.6 | (0.3-3.7) |
| 70 | 0.2 | (0.0-0.6) |  | 0.8 | (0.0-2.6) |
| 71 | 17.6 | (13.2-22.3) |  | 16.4 | (11.6-22.4) |
| 72 | 15.8 | (11.2-20.7) |  | 15.2 | (10.5-21.1) |
| 73 | 10.6 | (6.4-14.9) |  | 6.0 | (2.8-11.1) |
| 74 | 4.5 | (2.5-6.6) |  | 3.6 | (1.8-6.1) |
| 75 | 2.7 | (1.3-4.4) |  | 2.3 | (0.8-4.4) |
| 76 | 1.6 | (0.8-2.5) |  | 1.0 | (0.4-2.0) |
| 77 | 1.3 | (0.6-2.0) |  | 0.7 | (0.2-1.6) |
| 78 | 1.0 | (0.3-1.7) |  | 0.5 | (0.1-1.3) |
| 79 | 0.7 | (0.1-1.3) |  | 0.3 | (0.0-0.9) |
